# Modeling past and future spatiotemporal distributions of airborne allergenic pollen across the contiguous United States

**DOI:** 10.3389/falgy.2022.959594

**Published:** 2022-10-25

**Authors:** Xiang Ren, Ting Cai, Zhongyuan Mi, Leonard Bielory, Christopher G. Nolte, Panos G. Georgopoulos

**Affiliations:** ^1^ Environmental and Occupational Health Sciences Institute (EOHSI), Rutgers University, Piscataway, NJ, United States; ^2^ Department of Chemical and Biochemical Engineering, Rutgers University, Piscataway, NJ, United States; ^3^ Department of Environmental Sciences, Rutgers University, New Brunswick, NJ, United States; ^4^ Center for Environmental Measurement and Modeling, U.S. Environmental Protection Agency, Research Triangle Park, NC, United States; ^5^ Department of Environmental and Occupational Health and Justice, Rutgers School of Public Health, Piscataway, NJ, United States

**Keywords:** allergenic pollen, emissions and transport/fate model, climate change, CMAQ (Community Multiscale Air Quality model), CONUS (contiguous United States)

## Abstract

Exposures to airborne allergenic pollen have been increasing under the influence of changing climate. A modeling system incorporating pollen emissions and atmospheric transport and fate processes has been developed and applied to simulate spatiotemporal distributions of two major aeroallergens, oak and ragweed pollens, across the contiguous United States (CONUS) for both historical (year 2004) and future (year 2047) conditions. The transport and fate of pollen presented here is simulated using our adapted version of the Community Multiscale Air Quality (CMAQ) model. Model performance was evaluated using observed pollen counts at monitor stations across the CONUS for 2004. Our analysis shows that there is encouraging consistency between observed seasonal mean concentrations and corresponding simulated seasonal mean concentrations (oak: Pearson = 0.35, ragweed: Pearson = 0.40), and that the model was able to capture the statistical patterns of observed pollen concentration distributions in 2004 for most of the pollen monitoring stations. Simulation of pollen levels for a future year (2047) considered conditions corresponding to the RCP8.5 scenario. Modeling results show substantial regional variability both in the magnitude and directionality of changes in pollen metrics. Ragweed pollen season is estimated to start earlier and last longer for all nine climate regions of the CONUS, with increasing average pollen concentrations in most regions. The timing and magnitude of oak pollen season vary across the nine climate regions, with the largest increases in pollen concentrations expected in the Northeast region.

## Introduction

Airborne allergenic pollen, emitted from trees, weeds and grasses, is a major trigger of Allergic Airway Disease (AAD), affecting 5% to 30% of the population in industrialized countries (1–5). It has been estimated that pollen-related asthma emergency department visits across the contiguous United States (CONUS) will increase by 14% in 2090 under a high greenhouse gas emission scenario (6). Synergism of allergenic pollen with air pollutants such as ozone and particulate matter has been reported and can exacerbate the AAD of allergy sufferers (7–10). Pollen exposure also enhances susceptibility to respiratory viral infections, including SARS-CoV-2 (11–13).

The timing and magnitude of pollen seasons are determined by various biological and physical processes, where specific environmental conditions may affect single or multiple processes simultaneously. Temperature and precipitation have direct impact on plant phenology (14) and their changes can lead to shifts in pollen season onset and duration that diverge for spring-flowering and late-flowering taxa (15). Meteorological factors also affect the emission and transport of pollen grains. For instance, higher temperature, wind speed, and lower humidity favor pollen release (16); accumulated precipitation reduces airborne pollen *via* wet deposition (17). Furthermore, temperature and precipitation exert long-term effects on species distributions and alter pollen biology at multiple scales (18). There is evidence that plant populations are shifting northward where temperatures are cooler (19) as well as toward areas with greater water availability (20). Due to the complexities and interdependencies of the above processes, different observation-based studies, especially those at continental scales (e.g., the United States, Europe) over large time spans (10 to 30 years) across multiple taxa (21–23), have been conducted to assess the impacts of climatic drivers on pollen timing and levels. For instance, Anderegg et al. (22) report that pollen seasons started earlier by up to 20 days and pollen concentrations increased by 21% across North America, based on pollen observations (all taxa combined) from 1990 to 2018.

Observation-based methods analyze and associate historical trends and pollen indices with climatic factors, depending entirely on the data. However, pollen data are scarce in space and time - in the United States, only ∼80 pollen sampling stations (operated by a constellation of agencies and allergy clinics with different sampling methods) report their data to the National Allergy Bureau (NAB) of the American Academy of Allergy, Asthma, and Immunology (AAAAI) (24). In view of such limitations, process-based modeling of emissions and transport of allergenic pollens from multiple taxa are needed to isolate effects of climatic factors on the multiple underlying processes and to characterize their spatiotemporal distributions and associated potential consequences for public health. Previous studies of large scale emissions and long-range transport of pollen are summarized in Supplementary Table S1. Emission models can be constructed based on physics and phenology, to simulate emissions for the phenological phases of allergenic plants. The phenological phases are generally predicted in terms of functions of selected environmental factors. The transport and fate of pollen grains after release from the flower can be simulated using mesoscale meteorological models in conjunction with atmospheric transport models.

Recent studies modeling pollen emissions, transport and fate have focused on implementing mechanistic modeling approaches. A modeling framework incorporating the Weather Research and Forecast (WRF) model and the Community Multiscale Air Quality (CMAQ) model was used to study distributions of multiple allergenic pollens in southern California (25, 26). Jeon et al. (27) employed the emission model developed by Efstathiou et al. (28) to simulate oak pollen emissions in Southeast Texas and used the emissions in conjunction with the CMAQ model to predict oak pollen concentrations; however, the model underestimated oak pollen concentration and was not able to capture its peaks. Sofiev et al. (29–31) simulated birch pollen emissions and transport in Europe using the System for Integrated Modeling of Atmospheric Composition (SILAM). Mechanistic modeling systems have also been developed and applied to provide operational forecasts of ragweed pollen concentrations in Europe (32, 33). It should be noted that the above mechanistic models have mainly focused on short-term simulations of pollen concentrations for historical years and/or for small regions (except for SILAM). Large scale deterministic emission and transport/fate models need to be developed and evaluated for investigating the impacts of climate change on allergenic pollen distributions in the United States (34).

**Figure 1:**
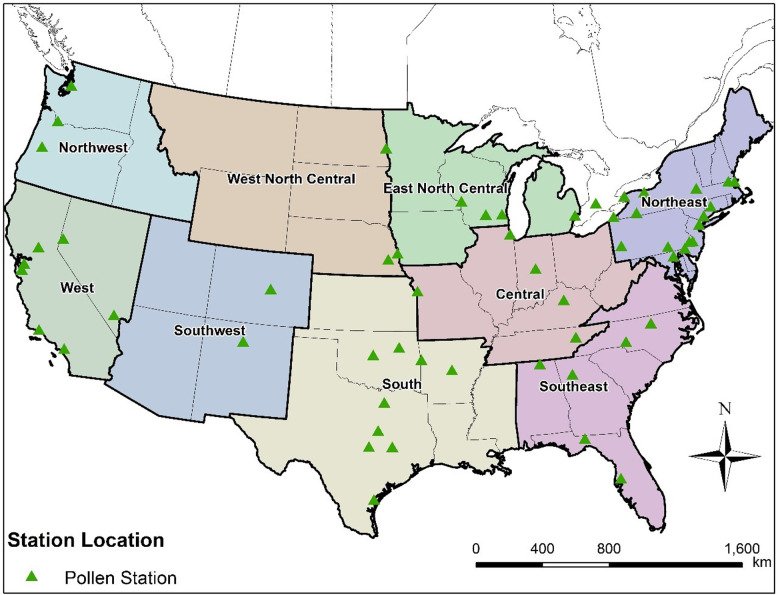
Distribution of the 58 studied pollen stations across the nine climate regions in the contiguous United States.

Our team has developed a platform for large scale airborne pollen modeling that incorporates our recent semi-mechanistic model for pollen emissions (16, 28, 35, 36). This new pollen emission model is coupled in the present study with our adapted version of the CMAQ model (Supplementary Figure S1) to simulate spatiotemporal distributions (hourly with horizontal resolution of 36 km × 36 km) of allergenic oak and ragweed pollen across the contiguous United States (CONUS) for both historical (2004) and future (2047) conditions. We selected oak (spring-flowering) and ragweed (summer/fall flowering) for analysis because ragweed is a widely distributed annual weed and its pollen is a leading cause of hay fever (37) while oak is one of the most allergenic tree species in the United States (38). The prevalences in NHANES III for positive test responses to 10 common allergens were 26.2% for short ragweed and 13.2% for white oak (39). Simulation results for 2004 were evaluated using observed pollen counts from the monitor stations of AAAAI-NAB operating across the CONUS during that year (Figure 1). Process analyses were conducted to investigate the contribution of different physical processes on airborne pollen concentrations as well as the effects of boundary conditions of airborne pollen concentration on simulated pollen concentration patterns and levels.

## Materials and methods

### Model configuration

The CMAQ-Pollen modeling system was established to produce hourly concentrations of airborne pollen at 36 km × 36 km spatial resolution across the CONUS (28, 36). The configurations of model components are listed in Supplementary Table S2. The meteorological inputs are derived from climatological simulations performed using the Community Earth System Model (CESM), which was downscaled using the WRF model (40, 41). In the present study, we performed simulations for a historical year (“2004”) and for a future year (“2047”); the two years were selected as representative of the climatological conditions in the beginning and middle of the 21st century. It should be noted that the meteorological information in the simulations was not intended to exactly “match” these actual meteorological years, since the climatological simulations were performed without assimilating daily local weather observations (40). The pollen transport and fate model CMAQ-Pollen was adapted from version 4.7.1 of the CMAQ modeling system (42, 43). Pollen grains are treated as inert coarse mode aerosol and physical properties such as density, diameter and diameter distributions and other related information (e.g., cutoff maximum aerosol diameter) of the coarse mode are incorporated in the relevant CMAQ modules (AERO5), so that the CMAQ-Pollen model can properly describe pollen dynamics. Specifically, pollen grains are modeled by adding to the existing coarse mode and assuming it is fully mixed with other coarse aerosols (e.g., agricultural and wind-blown dust, sea spray). The CMAQ modules describe diameter of each coarse aerosol *via* a lognormal distribution (44). Due to data limitation, we assume the means of pollen diameters to be fixed (oak = 28 μm and ragweed = 18 μm for both historical and future years) and set relevant parameters (such as pollen density) the same as those used in our emission model (16). The CMAQ-Pollen model was run for 2004 and 2047, covering the CONUS with 36 km × 36 km horizontal grid spacing, hourly temporal resolution, and 34 layers in the vertical direction. The vertical spatial resolution (layer height) varies by grid cell and time, noting that CMAQ uses a sigma vertical coordinate system (45) based on a pressure at the top boundary (the model top was set at 50 hPa). The simulation results for 2004 were evaluated using observed pollen counts from the 58 monitor stations of AAAAI-NAB (24) operating across the CONUS in 2004 (Figure 1). Methods for aeroallergen sampling may differ across monitor stations (46), with Rotorod samplers, Burkard samplers, and Kramer-Collins samplers, commonly used (47, 48). AAAAI-NAB guidelines require the samplers to be situated on an unobstructed rooftop at least one story above ground and each station must sample a minimum of 3 days per week with quality control (47). The meteorological conditions for 2047 correspond to a hypothetical high greenhouse gas emission scenario (Representative Concentration Pathway RCP8.5) (40).

### Emission model

The pollen emission flux in a cell of the modeling grid with area of *S_g_* was calculated through Eq. 1:

(1) $$F_{g}=F_{e}S_{g}P_{c}$$

where *P_c_* is the percentage of area coverage of allergenic plants in the corresponding modeling grid cell. The upward emission flux *F_e_* for a unit surface is derived using mass balance of pollen grain fluxes in the surroundings of allergenic plants (16); it is formulated using Eq. 2:

(2) $$F_{e}=\frac{q_{p}L_{d}L_{h}(K_{e}LAI+C_{r}K_{r}(1+LAI))}{1+v_{d}(1+LAI)/u_{*}}$$

where *q_p_* is the annual total emission flux. *L_d_* and *L_h_* are the daily and hourly flowering likelihoods, respectively. *K_e_* and *K_r_* (dimensionless) are the lumped meteorology adjustment factors for direct emission and resuspension fluxes, respectively. *LAI* is the leaf area index. *C_r_* is a proportionality factor relating the resuspension to direct emission flux. $u_{*}$ and *v_d_* are the characteristic velocity and the deposition velocity, respectively. All these terms on the right side of Eq. 2, can either be measured, or parameterized and approximated through measurable factors. Details of the derivation and parameterization of the emission model were presented in Cai et al. (16). The emission fluxes occur only in the first model layer, up to 60 meters above ground.

The future-year emissions were calculated using the meteorological factors for 2047, while the remaining parameters were the same as those in the historical emission model. Three main parameters (*K_e_*, *K_r_*, *L_d_*) contribute to emission differences between historical and future scenarios: both direct emission and resuspension processes are affected by temperature, wind speed and humidity [see Eqs 10–14 in our emission model (16)], and daily flowering process is affected by temperature and precipitation [see Table 1 in our phenology model (36)].

**Table 1:** Regional mean and standard deviation of changes between 2047 and 2004 (March-April) in mean and maximum hourly concentration, start date, season length and exceedance hours for oak pollen, estimated using the CMAQ-Pollen model. (mean ± standard deviation).

Climate region	Mean hourly (%)	Max hourly (%)	Start date (day)	Season length (day)	Exceedance hours (%)
Central	5.0 ± 43.4	−7.8 ± 23.9	−1.6 ± 4.0	−0.4 ± 1.0	2.2 ± 17.3
East North Central	−63.2 ± 24.7	−38.8 ± 73.9	3.1 ± 3.8	−1.5 ± 1.0	−32.7 ± 48.4
Northeast	89.7 ± 117.2	85.0 ± 193.6	−2.2 ± 1.2	−0.6 ± 1.2	31.6 ± 71.7
Northwest	−59.3 ± 39.4	−30.2 ± 54.9	2.9 ± 2.2	−2.1 ± 2.0	−60.7 ± 33.1
South	3.0 ± 3	18.6 ± 39.6	−1.4 ± 3.4	−0.2 ± 1.2	1.1 ± 24.9
Southeast	13.2 ± 37.7	5.2 ± 24.6	−7.7 ± 2.5	1.0 ± 1.4	9.4 ± 21.3
Southwest	5.9 ± 31.3	−0.2 ± 35.7	0.5 ± 3.3	1.4 ± 1.2	7.0 ± 30.1
West	−5.6 ± 31.7	3.0 ± 40.1	4.1 ± 2.8	−0.2 ± 1.1	−0.3 ± 22.9
West North Central	−52.4 ± 41.8	−45.8 ± 32.0	6.8 ± 2.8	−1.4 ± 1.1	−66.6 ± 30.1

### Physical processes governing transport of airborne pollen

The physical processes governing the transport and fate/removal of pollen grains from air include cloud processes, dry deposition, horizontal and vertical advection, and horizontal and vertical eddy (turbulent) diffusion (Supplementary Figure S1). The dry deposition process is incorporated in the vertical diffusion process as a flux boundary condition at the bottom of the model layer (49) and includes the effect of gravitational settling, which is the most important process governing deposition of coarse mode particles. Wet deposition is incorporated in the cloud processes, which include both in-cloud and below-cloud scavenging; wet deposition depends on the precipitation rate and on concentration in cloud water (44). Effects of convection on pollen transport are treated separately through modules of horizontal and vertical advection.

### Initial and boundary conditions

Simulation periods during 2004 and 2047 were selected to correspond to tree and weed pollen seasons. For oak, the simulations for the CONUS domain were run from 00:00 of March 1st through 23:00 of April 30th, 2004 and 2047. For ragweed, the simulations were run from 00:00 of August 1st through 23:00 of September 30th. March 1st and August 1st generally precede the earliest flowering day of oak and ragweed in the CONUS, respectively; therefore, the simulations were initialized with no existing pollen.

Boundary Conditions (BC) represent multi-layer concentration fields for grid cells surrounding the modeling domain, i.e., the eastern and western boundaries of simulation domain bordering the Atlantic and Pacific oceans, and northern and southern boundaries adjoining Canada and Mexico. To investigate the influence of BC on airborne pollen concentrations, two simulations on the CONUS domain were run for oak pollen for the period from March 1st to April 30th, 2004 by prescribing BC values for all 34 layers at the four lateral boundaries as (1) 0 (default) and (2) 10 pollen grains/m^3^ at each time step of the CMAQ-Pollen model. Because BC values are not sensitive to the simulated airborne pollen concentrations [concentration differences were below 2 pollen grains/m^3^ for most areas (Supplementary Figure S6)] and eastern and western boundaries are on the sea without pollen emissions, we set the BCs of pollen to zero.

### Evaluation of model performance

Since, as mentioned in Subsection “Model configuration”, meteorological inputs were downscaled from a global climate model without assimilating daily local weather observations (40), our hourly predictions for pollen concentrations were not expected to adequately capture fine-scale temporal variations. Therefore, we calculated “averages” and “upscaled” hourly predictions to reduce data variability, and evaluated model performance at seasonal and monthly temporal scales. It should be noted that such upscaling of the hourly pollen data can help reduce uncertainties and the biases associated with using different procedures for aerobiological monitoring (50). Correlation analysis of the observed seasonal mean pollen concentrations at individual pollen monitoring stations (Figure 1) with the corresponding simulated seasonal mean pollen concentrations was conducted with normalized pollen data ($X^{\prime }=(X-\bar{X})/s$, where $\bar{X}$ is the sample mean, and *s* is the sample standard deviation). The observed pollen concentrations at each monitor station are paired with the simulated pollen concentrations in the grid cell that contains the corresponding pollen monitoring station. The simulated pollen concentrations are derived from the simulated hourly concentrations in the model's lowest (surface) layer (i.e., layer 1) since observations of pollen counts are generally made near the ground. The model's lowest layer on average extends from 0 to 60 m above the ground. Estimates of fractional bias (FB) of simulated seasonal pollen counts (sum of daily pollen concentration) are reported as:

(3) $$FB_{i}=2\frac{SC_{\mathrm{Sim},i}-SC_{\mathrm{Obs},i}}{SC_{\mathrm{Sim},i}+SC_{\mathrm{Obs},i}}$$

where FB*_i_* is the fractional bias of simulated seasonal pollen counts at station *i*, SC_Sim*,i*_ is the simulated seasonal pollen count at station *i*, and SC_Obs*,i*_ is the observed seasonal pollen count at station *i*. Hit and false rates are common indices for evaluating the simulated daily pollen concentration. Procedures reported in the literature were followed to calculate the hit and false rates at three different concentration levels (32, 33), i.e. at 10, 50 and 100 pollen grains/m^3^, respectively. The details of the calculations are presented in the Supplementary Material.

### Process analysis

The Process Analysis preprocessor (PROCAN) was compiled together with our adapted CMAQ model to activate the process analysis function (42) in the CMAQ-Pollen modeling system. The process analysis was conducted to identify the contributions of each physical process on estimated airborne pollen concentrations. The physical phenomena incorporated in the process analysis include cloud processes, dry deposition, emissions, horizontal and vertical advection, and horizontal and vertical eddy (turbulent) diffusion. The process analysis was performed using the time series of simulated hourly concentrations of allergenic oak pollen during the pollen season in 2004 in the grid cell that contains the pollen monitoring station at the Atlanta Allergy and Asthma Clinic (coordinates: 33.97°N, 84.55°W). This area has an elevation of 366 m, annual mean temperature of 16.8 °C, and annual mean precipitation of 1,286 mm. We selected this station (with high annual production and peak concentration) as an example for process analysis; for other stations, contribution of each process to pollen prediction can be different, depending on meteorological conditions that vary both in space and time.

## Results

### Spatiotemporal patterns of ambient pollen concentrations

**Figure 2:**
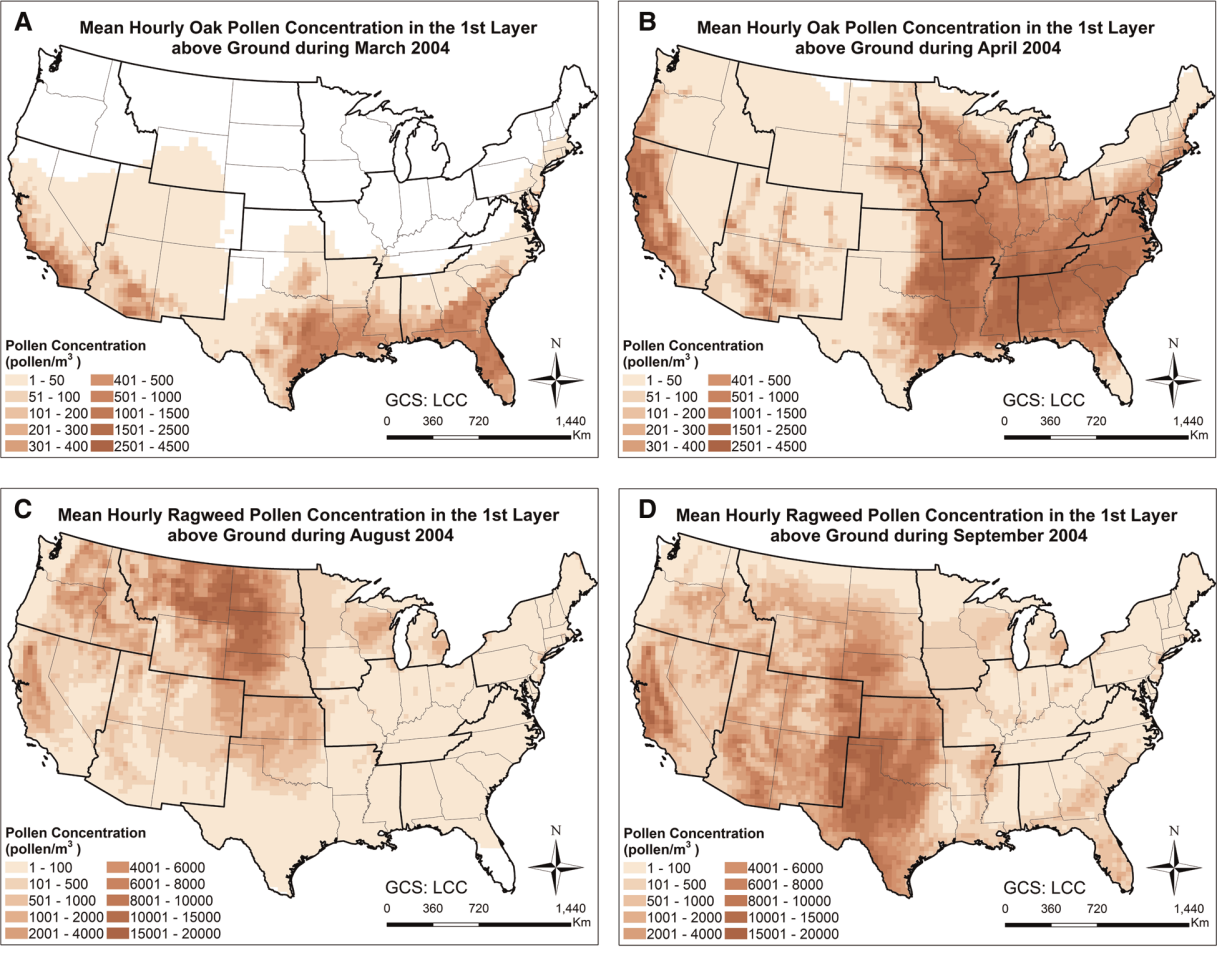
Spatial patterns of monthly mean concentrations of (**A**) oak pollen in March 2004; (**B**) oak pollen in April 2004; (**C**) ragweed pollen in August 2004; (**D**) ragweed pollen in September 2004, calculated using the CMAQ-Pollen model.

**Figure 3:**
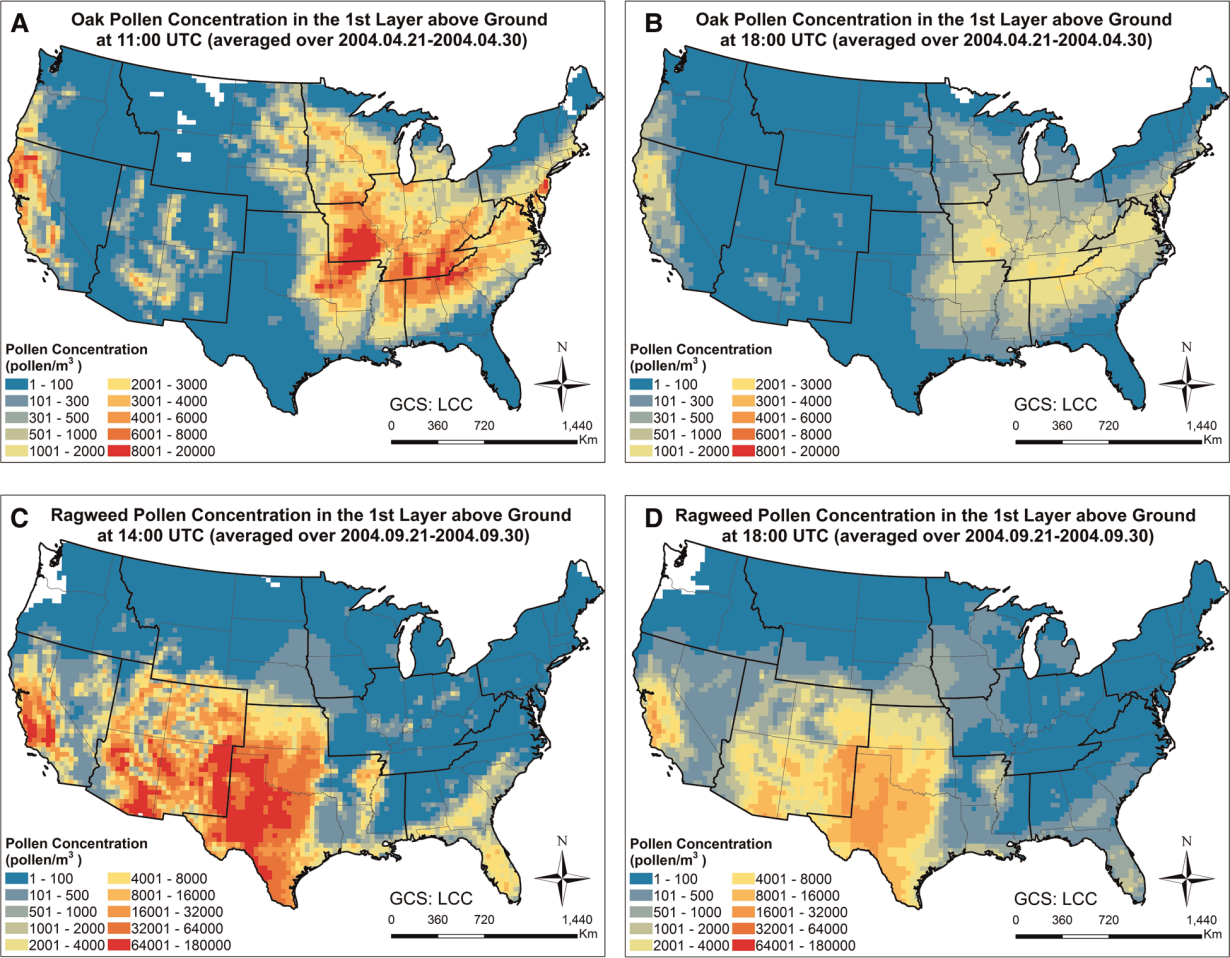
Time slices of spatiotemporal concentration profiles of (**A**) oak pollen at 11:00 UTC (averaged over Apr 21–Apr 30, 2004); (**B**) oak pollen at 18:00 UTC (averaged over Apr 21–Apr 30, 2004); (**C**) ragweed pollen at 14:00 UTC (averaged over Sept 21–Sept 30, 2004); (**D**) ragweed pollen at 18:00 UTC (averaged over Sept 21–Sept 30, 2004), calculated using the CMAQ-Pollen model.

In order to examine the spatiotemporal distribution patterns of the simulated airborne pollen, the monthly mean oak and ragweed pollen concentrations at ground level during their early and late flowering season are plotted in Figure 2. Oak pollen first appeared in the Southern CONUS in March, then observed in the Northern CONUS in April. The simulated maximum mean oak pollen concentration for 2004 is 4,500 pollen grains/m^3^. Ragweed pollen appeared first in the Northern CONUS in August and then shifted toward the Southern CONUS in September. The simulated maximum mean ragweed pollen was 2 × 10^4^ pollen grains/m^3^. Figure 3 displays simulated average oak and ragweed pollen concentrations for different hours of the day. These hourly concentration profiles (averaged over multiple days) indicate that our CMAQ-Pollen model can capture important spatiotemporal variations of airborne allergenic pollen. Consistent with known diurnal patterns, the oak pollen concentration in each cell of the modeling grid at 11:00 UTC is higher than that at 18:00 UTC (averaged over Apr 21–Apr 30, 2004), and the ragweed pollen concentration in each cell of the modeling grid at 14:00 UTC is higher than that at 18:00 UTC (averaged over Sept 21–Sept 30, 2004).

### Evaluation of model performance

**Figure 4:**
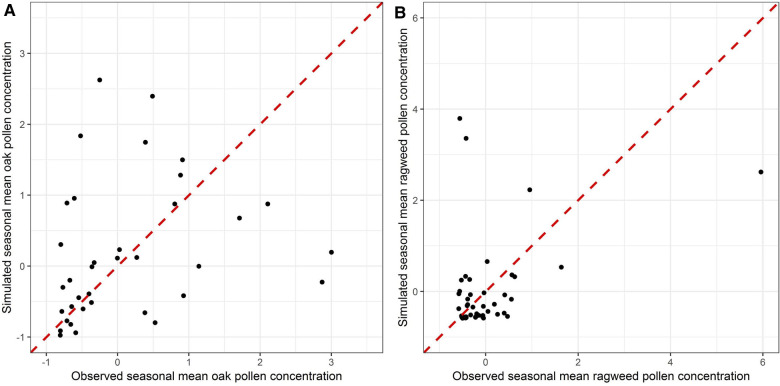
Scatterplots of normalized observed seasonal mean concentrations and simulated seasonal mean concentrations in 2004 for (**A**) oak and (**B**) ragweed pollen at pollen monitoring stations with available data.

**Figure 5:**
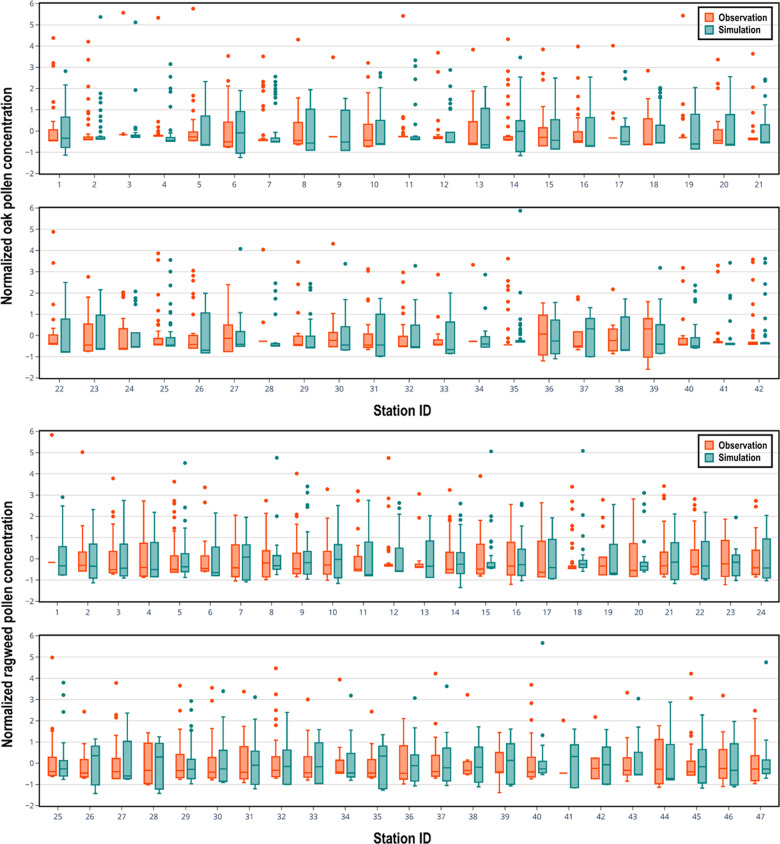
Seasonal box plots of normalized daily concentrations (simulated with the CMAQ-Pollen model) of oak pollen (top) and ragweed pollen (bottom) compared with observed pollen concentrations in 2004 at pollen monitoring stations. Boxes range from the 25th to 75th percentiles with the dark line denoting median and the dark dots denoting the outliers.

CMAQ has been extensively used to study the size, chemical composition, and atmospheric concentration of particulate matter, which is allocated into three modes (Aitken, accumulation, and coarse) in the aerosol modules, based on its size. Most CMAQ studies have focused on particles of the Aitken and accumulation modes (51–53). In the present study, pollen grains are treated as inert coarse mode aerosol and the performance of CMAQ in predicting pollen grain numbers is evaluated for the first time. As shown in Figure 4, there is encouraging consistency between observed seasonal mean concentrations and the corresponding simulated seasonal mean concentrations for both oak and ragweed pollens. The Pearson correlation coefficient is 0.345 (*p*-value is 0.0252) for oak pollen based on available data from 42 monitoring stations, and 0.399 (*p*-value is 0.0055) for ragweed pollen based on data from 47 monitoring stations. The data points for oak pollen are evenly distributed around the 45-degree line. Three ragweed pollen monitoring stations have larger deviations, compared to other stations, which our model was not able to capture. The statistical distributions of the daily simulated pollen concentrations at each pollen monitoring station during the pollen season, compared with corresponding observation data are shown in Figure 5. For each pollen monitoring station, similar distributions of simulation results and observation data indicate reasonably good model performance. Our model was able to capture the distribution patterns of observed pollen concentration for most of the stations. For oak pollen, the model was also able to simulate the extreme data points at the stations with high concentration outliers.

**Figure 6:**
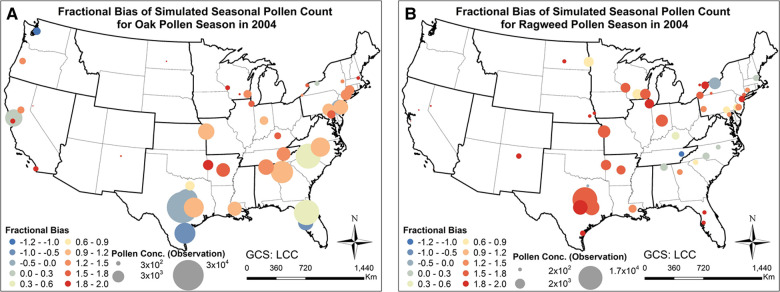
Fractional biases of pollen concentrations calculated with the CMAQ-Pollen model for 2004 in the CONUS. (**A**) Fractional bias of seasonal oak pollen counts; (**B**) Fractional bias of seasonal ragweed pollen counts.

Figure 6 shows the fractional bias of the simulated seasonal pollen count. The fractional bias for seasonal oak pollen count was in most cases greater than 0, indicating overestimation of the pollen concentration. The fractional bias for ragweed pollen concentration was also greater than 0 for most stations, again suggesting overestimation of observed levels. The fractional bias exhibits spatial autocorrelation to some degree: for instance, we detected spatial autocorrelations of estimated pollen onset and duration parameters that will affect pollen prediction (36). However, the model was able to capture the variation of the pollen observations as shown in the correlation analysis summarized in Figure 4. Hit and false rates are metrics for checking whether the simulated and observed exceedances are consistent and co-located. Supplementary Figures S3, S4 present the hit rates and false rates for predicted and observed daily oak and ragweed pollen concentrations for three pollen levels at the studied stations during 2004 across the CONUS. The average hit rates for airborne oak pollen levels of 10, 50 and 100 pollen grains/m^3^ were 81.9%, 74.1% and 68.6%. The average hit rates for airborne ragweed pollen levels of 10, 50 and 100 pollen grains/m^3^ were 85.7%, 72.1% and 72.0%. This indicates that the observed exceedances of the three thresholds were mostly correctly predicted by the present modeling system for pollen emission and transport/fate. The false rates for an airborne oak pollen level of 10 pollen grains/m^3^ were between 0% and 30% for most of the studied stations, but the false rate increased for levels of 50 and 100 pollen grains/m^3^; the false rates for ragweed were over 30% for most stations, which indicates that the CMAQ-Pollen model overestimated the ragweed pollen concentration. Process analysis results shown in Supplementary Figure S5 indicate that different processes can play their roles in determining pollen concentrations, depending on the meteorological conditions and emissions that vary diurnally and seasonally.

### Impact of climate change on allergenic pollen

**Figure 7:**
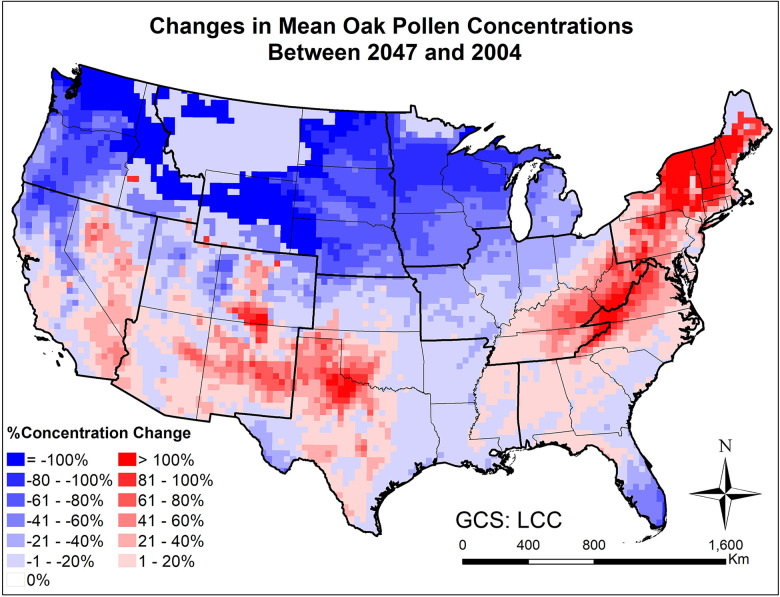
Changes in mean March-April oak pollen concentrations from beginning (2004) to mid-21st century (2047) estimated for the RCP8.5 climate change scenario using the CMAQ-Pollen model.

Our pollen emission and transport/fate model was also used to study potential climate change impacts on attributes of allergenic pollen seasons in the CONUS. Supplementary Figures S8, S9 show the spatial distribution of the mean and maximum hourly concentrations of oak and ragweed pollen in 2004 and 2047. The mean and maximum concentrations vary substantially across the nine climate regions of the CONUS. For oak pollen, the highest mean and maximum concentrations occurred in the Central, Southeast and South regions. For ragweed pollen, the highest mean and maximum hourly concentrations occurred in the West North Central, South, and Southwest regions. The simulated mean hourly concentrations of oak pollen ranged from 1 to 2,442 pollen grains/m^3^ in 2004 and from 1 to 2,360 pollen grains/m^3^ in 2047; the simulated maximum hourly concentrations of oak pollen varied from 12 to 27,306 pollen grains/m^3^ in 2004 and from 3 to 29,175 pollen grains/m^3^ in 2047. The decrease in mean hourly concentration and increase in maximum hourly concentration can be explained by the fact that the dispersion of data will increase with greatest minimum values in many locations. For instance, significant increases of standard deviation of hourly oak pollen concentrations were observed across the State of Idaho, Oregon and Nevada and across much of the region in Kansas (Supplementary Figure S10), and these regions had decreasing mean (Figure 7) and increasing maximum (Supplementary Figure S15A) hourly oak pollen concentrations. This suggests that the variability of pollen season can increase across large areas of the CONUS under changing climate. The simulated mean hourly concentrations of ragweed pollen ranged from 1 to 11,567 pollen grains/m^3^ in 2004 and from 1 to 12,187 pollen grains/m^3^ in 2047; the simulated maximum hourly concentrations of ragweed pollen varied from 23 to 3.6 × 10^5^ pollen grains/m^3^ in 2004 and from 76 to 3.7 × 10^5^ pollen grains/m^3^ in 2047.

Supplementary Figures S11, S12 present the simulated start date and season length of oak and ragweed pollen season in 2004 and 2047. The oak pollen season in 2004 and 2047 starts in March in the Southern US, and in April in the Northern US, while the ragweed pollen starts from the Northern US in August, and then shifts toward the Southern US in September. The oak pollen season length ranged from 12 to 46 days in 2004 and 10 to 46 days in 2047, and the ragweed pollen season length varied between 30 and 57 days in 2004 and between 32 and 58 days across the CONUS. Supplementary Figures S13, S14 display the number of hours during which oak and ragweed pollen concentration exceeded the threshold values (13 pollen grains/m^3^ for oak and 30 pollen grains/m^3^ for ragweed) in 2004 and 2047 across CONUS. The threshold values were selected based on clinical symptoms of allergic disease in sensitive patients (5, 54, 55). The oak pollen exceedances ranged from 0 to 1,462 h in 2004 and from 29 to 1,436 h in 2047, with the highest numbers appearing in the South, Southeast, Southwest and the West climate regions. The ragweed pollen exceedances ranged from 0 to 1,208 h in 2004 and from 29 to 1,234 h in 2047, with the highest numbers in the West North Central, the Southwest, West, and the Northwest climate regions.

**Figure 8:**
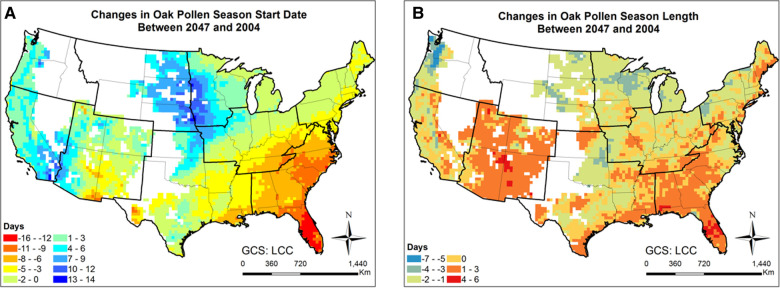
Changes in oak pollen season between 2047 and 2004 estimated for the RCP8.5 climate change scenario using the CMAQ-Pollen model. (**A**) Changes in oak pollen season start date between 2047 and 2004; (**B**) Changes in oak pollen season length between 2047 and 2004. Data were mapped only on cells in which the area coverage of oak trees is greater than zero.

Figures 7, 8 and Supplementary Figure S15 present the changes of five pollen indices, specifically, mean hourly concentrations, start date, season length, maximum hourly concentrations, and exceedance hours for oak pollen from 2004 to 2047. The mean and standard deviation of the changes in these five oak pollen indices for each climate region are summarized in Table 1, showing that the impact of climate change on oak pollen varies substantially across the nine CONUS climate regions. The mean and maximum hourly concentrations of oak pollen are predicted to increase in the Northeast, South and Southeast regions, but to decrease in the Northwest, East North Central, and West North Central regions. The Northeast region is estimated to experience on average the highest increase in mean and maximum hourly concentrations for oak pollen. The oak pollen season was estimated to start earlier in the Central, Northeast, South and Southeast regions. Furthermore, the oak pollen season length was estimated to shorten by 1–2 days for most regions, except for the Southeast and Southwest regions. The number of hours during which the oak pollen concentrations exceed a threshold value of 13 pollen grains/m^3^ is estimated to increase the most in the Northeast region, by 31.6%.

**Figure 9:**
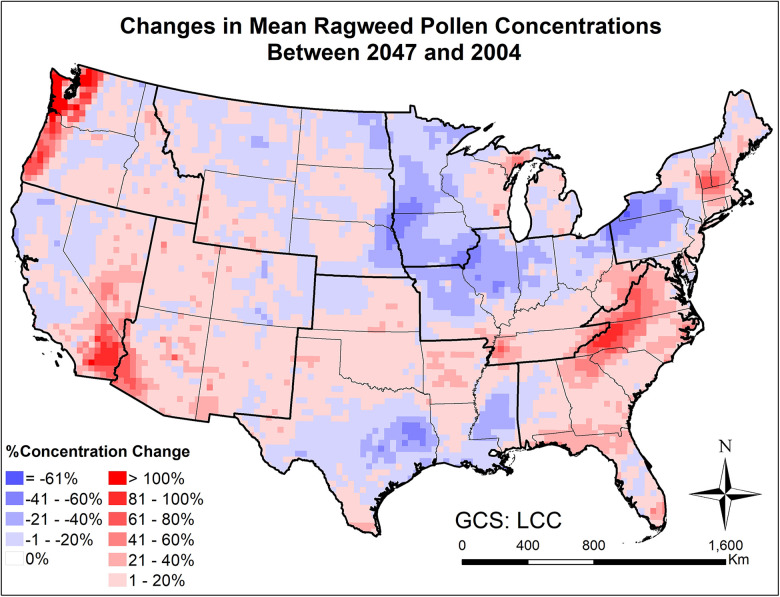
Changes in mean August-September ragweed pollen concentrations from beginning (2004) to mid-21st century (2047) estimated for the RCP8.5 climate change scenario using the CMAQ-Pollen model.

**Figure 10:**
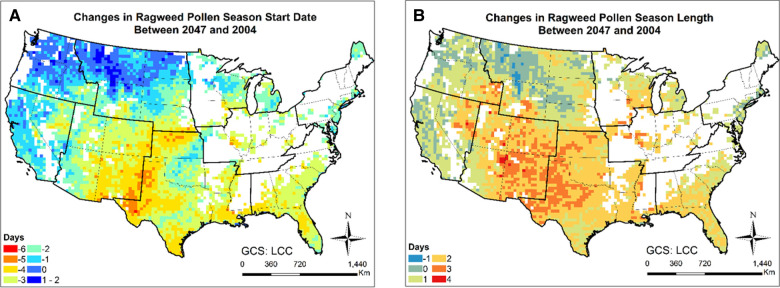
Changes in ragweed pollen season between 2047 and 2004 estimated for the RCP8.5 climate change scenario using the CMAQ-Pollen model. (**A**) Changes in ragweed pollen season start date between 2047 and 2004; (**B**) Changes in ragweed pollen season length between 2047 and 2004. Data were mapped only on cells in which the area coverage of ragweed is greater than zero.

Figures 9, 10 and Supplementary Figure S16 present the changes in the five pollen indices for ragweed pollen from 2004 to 2047. The regional mean and standard deviation of the changes in the five ragweed pollen indices are summarized in Table 2. The response of ragweed pollen to climate change also varies substantially across the nine climate regions. The mean and maximum hourly concentrations of ragweed pollen are predicted to increase significantly in the Northwest, Southeast, Southwest and West regions. The ragweed pollen season is estimated to start 1–3 days earlier, with a longer pollen season in all nine climate regions. The number of hours during which the ragweed pollen concentrations exceed a threshold value of 30 pollen grains/m^3^ are estimated to increase by 1.2%–34.3% in six of the nine climate regions, while there was a decrease in the Central, East North Central and West North Central regions. Of course, it should be recognized that these potential changes correspond specifically to one, high greenhouse gases emissions scenario (RCP8.5) and should only be interpreted in that context.

**Table 2:** Regional mean and standard deviation of changes between 2047 and 2004 (August-September) in mean and maximum hourly concentration, start date, season length and exceedance hours for ragweed pollen, estimated using the CMAQ-Pollen model. (mean ± standard deviation).

Climate region	Mean hourly (%)	Max hourly (%)	Start date (day)	Season length (day)	Exceedance hours (%)
Central	−2.1 ± 23.3	−1.2 ± 38.1	−2.8 ± 0.7	1.8 ± 0.6	−0.6 ± 28.9
East North Central	−12.5 ± 18.5	2.3 ± 32.3	−2.0 ± 0.8	1.4 ± 0.7	−19.7 ± 9.7
Northeast	−0.6 ± 25.1	0.1 ± 53.5	−1.8 ± 0.7	0.7 ± 0.5	11.9 ± 26.9
Northwest	19.3 ± 40.7	18.6 ± 50.7	−0.7 ± 0.9	0.9 ± 0.8	34.1 ± 195.1
South	0.5 ± 14.3	−5.3 ± 24.1	−3.3 ± 1.0	2.0 ± 0.6	1.8 ± 9.7
Southeast	22.7 ± 21.0	12.4 ± 43.2	−2.9 ± 0.9	1.4 ± 0.6	34.3 ± 36.5
Southwest	10.7 ± 14.6	3.7 ± 33.7	−3.1 ± 0.9	2.1 ± 0.7	1.2 ± 4.8
West	11.4 ± 23.2	9.0 ± 33.6	−1.5 ± 0.8	1.0 ± 0.8	3.9 ± 6.6
West North Central	−2.5 ± 12.0	2.7 ± 25.2	−1.1 ± 1.2	0.8 ± 0.8	−3.8 ± 8.3

## Discussion

Large scale mechanistic pollen forecasting under changing climatic conditions has been rare; this is one of few efforts aiming to develop and apply process-based modeling for species with highly allergenic pollen on a continental scale. The modeling results indicate that climate change is expected to increase airborne pollen loads by mid-century across the CONUS, while more significant increases will occur for areas where pollen is historically uncommon. For instance, over 40% increase in mean oak pollen concentration will occur across much of the Northeast and Southwest regions (Figure 7), where relatively low levels (<200 pollen grains/m^3^) were observed at the beginning of the century; over 20% increase in mean ragweed concentration will occur across much of the Northeast and Southeast regions (Figure 9), where relatively low levels (<500 pollen grains/m^3^) were observed at the beginning of the century. This finding is basically consistent with the study of long-term predictions of ragweed in Europe (56) that considered different plant invasion scenarios, and reported that sensitization to ragweed will more than double by mid-century. In the United States, a recent study has also indicated potential northward expansion of ragweed due to climate change (37).

Synergistic effects of allergenic pollen are important metrics for assessing the severity of pollen-related allergen exposures. Our model can detect both consistent (e.g., Northeast) and opposite (e.g., Southeast) patterns for oak and ragweed changes across different climate regions. Climate change in general will drive higher pollen loads and lengthen season durations as a consequence of the increasing temperature and CO_2_ levels (18). However, these impacts may be modulated by other, species-specific, factors that could offset or even reverse the predicted patterns (57). For instance, some tree species such as birch (58) and oak (59) could suffer the declining of their populations, as they are sensitive to increased temperatures and summer droughts. There have been observation-based studies conducted to explore taxa-specific effects for monitor locations at continental scale (21, 23), while mechanistic process-based models used to analyze future pollen patterns for multiple taxa are absent (34). A recent study employed a “climate-flexible” emission model (34) to project pollen emissions for 13 of the most prevalent airborne pollen taxa over the United States and compared the climate-driven changes in pollen timing and magnitude for all taxa (60). It should be noted that the study used scaling factors (such as the precipitation factor) to adjust pollen emission flux and quantified some “averaged” effects for multiple underlying processes; furthermore, dispersion and transport of pollen in the atmosphere were not investigated.

Importance of atmospheric transport has been well recognized for pollen forecasts (61). For instance, 7-year-long (2005–2011) SILAM model simulations were performed for ragweed across Europe, highlighting the important role of long-range transport in forming the high concentration patterns (32). Our modeling results have shown that the airborne pollen distribution patterns (Figure 2) generally follow the emission patterns in Figure 4 of Cai et al. (16), consistent with the findings in Kurganskiy et al. (17); furthermore, long-range transport has significant effects on climate-driven concentration changes at regional scales. Areas such as Nevada and northern Texas, without oak coverage (blank areas in Figure 8) were predicted to have 20%–100% increase in airborne oak pollen by mid-century; areas such as Massachusetts and Virginia, without ragweed coverage (blank areas in Figure 10) were predicted to have 20%–80% increase in airborne ragweed pollen by mid-century. Note that the same land use and plant coverage were applied in the present study to simulate historical and future concentration patterns. For areas with large species coverage, the emission process plays a dominant role in airborne pollen concentrations; while contributions of other processes (such as wind-driven horizontal advection) vary substantially, depending on meteorological conditions in specific areas (Supplementary Figure S5). For areas without relevant species coverage, emissions become zero and the atmospheric transport processes drive the airborne pollen concentrations.

The impacts of climate change on airborne allergenic pollen are complex, relating to multiple processes with large uncertainties and interdependencies. Apart from the consequence of increasing temperature and CO_2_ levels (18), other climate conditions such as alterations to precipitation patterns and rise in sea levels may also influence species distribution and pollen production (14, 62). It should be noted that the present study is mainly focused on quantifying important “isolated” effects of climatic factors (including temperature, precipitation, humidity and wind) on emission and transport of highly allergenic pollen (oak and ragweed) across the CONUS. Future changes in land use and other relevant factors should be further considered and incorporated in modeling platforms. As an example, Hamaoui-Laguel et al. (63) developed a comprehensive and integrated process-based modeling framework that incorporates phenology, plant population dynamics, pollen production, release and atmospheric transport to assess future changes in airborne pollen concentration (daily); the framework was further used for long-term forecasting and quantitative analyses of climate change impacts on ragweed pollen allergy in Europe (56).

Previous studies modeled impacts of climate change generally based on long-term predictions of pollen concentrations at continental or global scales (56, 60). Extrapolation for both past and future behaviors can be improved by calculating the average of simulations for multiple years; this is particularly true for empirical and simplified prognostic models (60). By comparison, process-based models are able to isolate effects of specific factors and are more robust and interpretable (Supplementary Figure S5), due to the fact that mechanistic patterns extracted from underlying processes can be easily extended to both past and future conditions. It should be recognized that the present study produced process-based modeling outcomes for one historic (2004) and one future (2047) year in order to characterize plausible changes in pollen concentrations from beginning to mid-century under a specific (RCP8.5) scenario, without accounting for interannual variability. Extensive work is ongoing to extend the CMAQ-Pollen model for applications involving multi-year forecasting of long-term pollen concentrations in the context of climate change.

## Conclusions

A CONUS-wide CMAQ-Pollen modeling system incorporating pollen emissions and transport has been developed to simulate spatiotemporal distributions of allergenic oak and ragweed pollens. Model performance was evaluated through correlation analysis, statistical distributions, hit and false rates, and fractional bias using observed pollen counts at monitor stations across the contiguous United States (CONUS) in 2004. The modeling results revealed encouraging consistency with observed pollen concentrations and the model captured important diurnal/seasonal patterns and spatial variations for both oak and ragweed pollens. Five pollen indices (mean hourly concentrations, maximum hourly concentrations, start date, season length, and exceedance hours) were calculated and compared for each pollen species across the nine climate regions of the CONUS for 2004 and for 2047. It was estimated that ragweed pollen season will start earlier and last longer under the RCP8.5 scenario for all the nine climate regions, with increasing average pollen concentrations in most regions. The response of the oak pollen season to climate change varies across the nine climate regions, with the largest increase in pollen concentration predicted for the Northeast region. We found that long-range transport had significant effects on climate-driven concentration changes, particularly for areas where pollen was historically uncommon. These effects were influenced by multiple underlying processes that can be isolated and interpreted *via* process analysis and integration. Additional quantitative studies incorporating modeling efforts that will consider alternative scenarios regarding future patterns of vegetation in long-term forecasting of airborne allergenic pollen, are needed to expand and to improve the understanding of climate change impacts on allergic disease.

## Data Availability

Model parameters and outputs are available upon request. Requests to access these datasets should be directed to panosg@ccl.rutgers.edu.
