# Supplementary material for: Modeling past and future spatiotemporal distributions of airborne allergenic pollen across the contiguous United States
Source: Front Allergy. 2022 Oct 25;3:959594. doi: 10.3389/falgy.2022.959594 (PMC9640548; doi:10.3389/falgy.2022.959594)
Supplement: Supplementary file 1 [file Datasheet1.pdf]

Supplementary Material

Calculation of hit and false rates:

The simulated daily pollen concentrations, and sum of daily pollen concentrations during the pollen season (hereafter referred as seasonal count) in 2004 were first paired with the corresponding observations. For example, the observed daily pollen concentrations at a monitor station was paired with the simulated daily pollen concentrations in a grid cell that contains the corresponding pollen monitoring station; similar pairings were performed for seasonal count. As shown in Eq. (A.1):

$$C(Day,i,j)=\frac{\sum_{hr\in Day}C(hr,1,i,j)}{24} \quad \text{(A.1)}$$

the simulated daily pollen concentration at a given day in a grid cell (i, j), $C(Day,i,j)$ is defined as the daily average concentrations derived from the simulated hourly concentrations on the model's lowest layer (i.e., layer 1) because observations of pollen counts are generally made near the surface. The model's lowest layer is on average located between 0 and 60 m above the ground.

Hit and false rates were calculated for evaluation of the simulated daily pollen concentration. Procedures in the literature were followed to calculate the hit and false rates at three different concentration levels (1, 2), which are 10, 50 and 100 pollen grains/m^3, respectively. Supplementary Table 3 lists the confusion matrix representing the number of cases of True Positive (TP), True Negative (TN), False Positive (FP) and False Negative (FN) of observed and predicted pollen concentrations for a given concentration level. The hit rate (H_i) and false rate (F_i) for a given pollen concentration level C_{Li} are defined using Eq. (A.2):

$$\begin{cases} H_i=\dfrac{TP_i}{TP_i+FN_i} \\ F_i=\dfrac{FP_i}{TP_i+FP_i} \end{cases} \quad \text{(A.2)}$$

Both hit and false rates are defined based on observations and simulations day by day. If both the observation and simulation for a given day at a given station are equal to or higher than threshold value level *i* (i.e., C_{Li}), it is counted as a hit (i.e. True Positive) for level *i* at this station. If the observation is below C_{Li} and the simulation is equal to or greater than C_{Li}, it is counted as a false (i.e., False Positive). The hit rate indicates among the observed airborne concentrations greater than or equal to C_{Li}, how many are correctly predicted. The false rate indicates among the predicted airborne concentrations greater than or equal to C_{Li}, how many are falsely predicted.

Process analysis

Supplementary Figure 5 presents the contributions of advection, eddy diffusion, dry deposition, emission and cloud processes on the hourly oak pollen concentrations between local time 00:00 UTC April 1 to 23:00 UTC April 10, 2004 in Atlanta, Georgia. The dry deposition, emission and vertical eddy diffusion are the dominant processes determining ambient concentrations of oak pollen. The emission process continuously released pollen grains into the air, following a regular diurnal pattern. The majority of ambient pollen grains were removed from the air through dry deposition. Generally, the pollen concentration near the surface represents a balance between emission and dry deposition. However, vertical eddy diffusion may dominate the transport of ambient pollen grains when there is strong turbulent atmospheric movement. For example, pollen grains may be lifted up by turbulence at neighboring locations (e.g., during a frontal passage) and subsequently transported horizontally to Atlanta where vertical diffusion could bring them down to the lowest layer under special weather conditions, thus increasing the pollen concentrations in the lowest layer. The cloud processes also played an important role through in-cloud and below-cloud scavenging during rainy time (red line between April 6 and April 8 in Supplementary Figure 5). Hence, depending on the meteorological conditions and emissions, both varying diurnally and seasonally, different processes can play important roles in determining the magnitude of pollen concentrations near the surface, and influence the model prediction skill.

Influence of boundary conditions

Supplementary Figure 6 displays the differences in ambient oak pollen concentrations due to different boundary conditions. In the majority of the grid cells, the mean oak pollen concentration seem not to be substantially influenced by the boundary conditions. In some areas of the west coast, such as California, Nevada, Arizona, Utah, Oregon and Washington, the mean oak pollen concentration increased by 1-2 pollen grains/m^3 because of the changes in boundary conditions. These areas appear to have relatively low oak area coverage and low emissions, so transport of pollen from the boundaries may be a dominant source of oak pollen in those regions. In the current study, the simulated pollen concentrations were only mapped within the CONUS boundaries, which are different from the model boundaries. An early study in some regions of California has reported that dynamic BCs had barely improved the model performances, and that perturbations in emissions significantly influenced the simulated pollen concentrations (3). Further investigations are needed to fully characterize the impact of BC on simulated pollen concentrations.

Uncertainty analyses

There are substantial uncertainties associated with each of the components and modules of our modeling system for pollen emissions and transport. For each of the model components and its modules, the uncertainty may result from model formulation, parameters and input data. Supplementary Table 4 presents the sources of uncertainties for each component and module in the current modeling system and the corresponding general assessments. In the current study, focused efforts have been made to identify and reduce the uncertainties in each of the model components and modules based on different methods.

For the meteorology simulations employing the WRF model, quality control measures have been applied by the collaborating group at the U.S. Environmental Protection Agency (EPA) to evaluate the quality of the archived meteorology data (4, 5). The pollen counts themselves may also be subject to large uncertainties (6). The observed pollen counts from NAB-AAAAI stations were examined carefully with respect to consistency with quality control measures to ensure data quality (7, 8).

For our pollen emission model, global sensitivity analysis was conducted to identify sensitive and interacting input parameters based on Morris' design (6, 9). The values of highly sensitive and interacting parameters were carefully chosen from the literature or parameterized using literature data. Many iterations of the emission model have been performed to ensure the consistency and quality of the simulated pollen emission data. For the pollen transport model, process analysis has been conducted to identify the contributions of each relevant physical process on the airborne pollen concentrations. The transport model was not nudged toward the observed pollen counts.

Supplementary Table 1. Modeling studies focusing on large-scale emissions and long-range transport of pollen (modified from Table S1 of Cai, Zhang (9)).

Study Location	Resolution, Layers	Plant Taxon	Emissions Model	Vegetation Data, Model	Meteorology Data, Model	Transport Model	Reference
Japan	10x10 km, 1	Cedar	Meteorological parameterization	Forest maps and remote sensing	AMeDAS weather stations	Gaussian model	Kawashima and Takahashi (10)
Germany	4x4 km, 35	Hazel, Alder	Empirical model	Forest maps	KAMM 3-D	DRAlS CTM	Helbig, Vogel (11)
St. Louis county, USA	12x12 km, 8	Oak	Uniform diurnal profile	BELD3.1 database	MM5, Eta 3-D	HYSPLIT	Pasken and Pietrowicz (12)
Finland	1x1 km, 1	Birch	Aerobiology observations	CORINE, PELCOM, Forest surveys	SILAM	SILAM	Sofiev, Siljamo (13)
Germany	500x500 m, 32	Oak	Meteorological parameterization	Forest maps	METRAS	METRAS	Schueler and Schlünzen (14)
Switzerland	7x7 km, 40	Birch	Empirical model	National Forest Inventory	COSMO-ART	COSMO-ART	Vogel, Pauling (15)
NorthEastern USA	12x12 km, 22	Birch, Ragweed	Empirical model	BELD3.1, PLANTS, MODIS LAI	MM5	CMAQ	Efstathiou, Isukapalli (16)
California, USA	12x12 km, 4x4 km, 29	Multiple Taxa	STaMPS	NASS of USDA, tree inventory	WRF	CMAQ	Zhang, Duhl (3); Duhl, Zhang (17)
Europe	0.25°x0.25°, 40, 74	Birch	Probabilistic model	CORINE, PELCOM, Forest surveys	ECMWF, HIRLAM	SILAM	Siljamo, Sofiev (18); Sofiev, Siljamo (19)
Central Europe	7x7 km, 40	Ragweed	Empirical model	Empirical model	COSMO-ART	COSMO-ART	Zink, Vogel (2)

Europe	0.25ºx0.25º, 8	Ragweed	Empirical model	Ecological model	ECMWF	SILAM	Prank, Chapman (1)
Europe	Multiple resolution and layers	Birch	Probabilistic model	CORINE, PELCOM, Forest surveys	ECMWF, WRF	Multiple Ensemble Members	Sofiev, Berger (20)
Texas, USA	4x4km, 27	Oak	Empirical model	BELD3.1, PLANTS, MODIS LAI	WRF	CMAQ	Jeon, Choi (21)
USA	36x36 km, 34	Oak, Ragweed	Mechanistic model	BELD3.1, Empirical model	WRF	CMAQ	This study

Supplementary Table 2. Configuration of the meteorology, emission and transport model for studying distributions of airborne allergens.

	Model	Resolution, Layers	Period	Domain	References
Meteorology	WRF v3.4.1	36x36 km, hourly, 34	2004, 2047	Contiguous US	(4, 5)
Emission	Semi-mechanistic model	36x36 km, hourly, 1	2004, 2047	Contiguous US	(9)
Transport & Fate	Adapted CMAQ v4.7.1	36x36 km, hourly, 34	2004, 2047	Contiguous US	Current study

Supplementary Table 3. Confusion matrix for calculating hit rate and false rate.

Greater or equal to concentration level *i* (i.e., $\geq C_{Li}$)		Observation	
		True	False
Prediction	True	TP_i	FP_i
	False	FN_i	TN_i

Supplementary Table 4. Sources of uncertainties for each component and module in the deterministic modeling system, and the corresponding general treatments.

Uncertainty Source		Model Formulations	Model Parameters	Input Data	Notes
**Emission Model**	Flowering likelihood	Relevant	Relevant	Relevant	(9)
	Vegetation coverage	Relevant	Relevant	Relevant	
	Start date and season length	Relevant	Relevant	Relevant	
	Meteorology adjustment factors	Relevant	Relevant	Not relevant	
**Transport & Fate Model**	Advection	Relevant	Relevant	Not relevant	Adapted from CMAQ v4.7.1
	Diffusion	Relevant	Relevant	Not relevant	
	Cloud process	Relevant	Relevant	Not relevant	
	Dry deposition	Relevant	Relevant	Not relevant	
	Initial, Boundary conditions	Not relevant	Not relevant	Relevant	
**Meteorology Model**	Microphysics	Relevant	Relevant	Relevant	(4, 5)
	Long and short wave radiation	Relevant	Relevant	Relevant	
	PBL scheme, land scheme, etc.	Relevant	Relevant	Relevant	
	Initial, Boundary conditions	Not relevant	Not relevant	Relevant	
	Ensemble members	Relevant	Relevant	Relevant	
**General Treatment**		New model methods or modified formulations	Global uncertainty, sensitivity analyses	Statistical techniques to clean data	

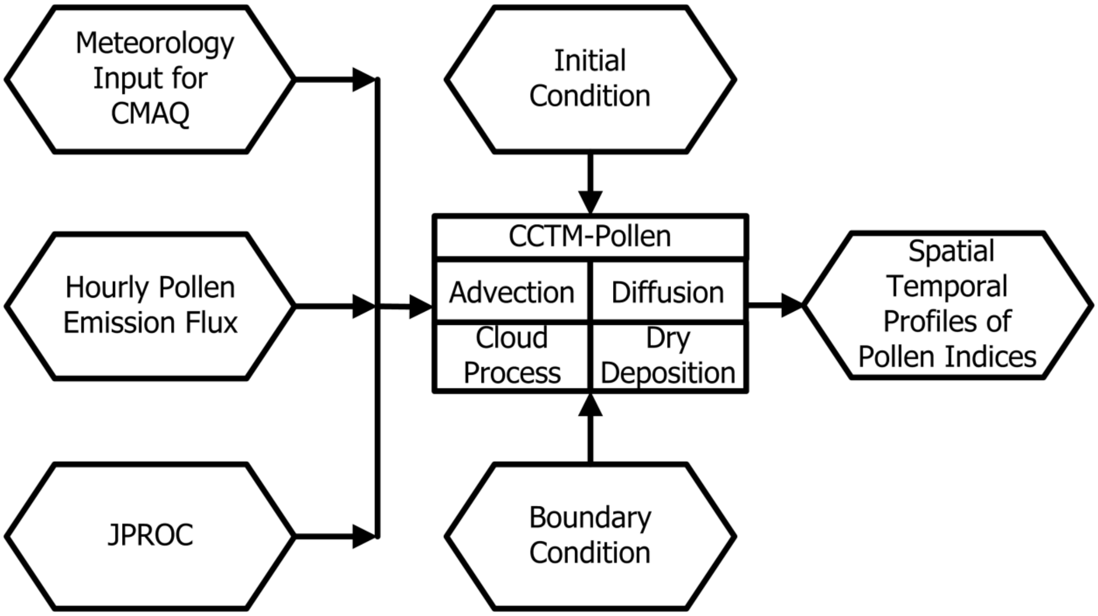

Supplementary Figure 1. Schematic diagram of pollen transport model. The model was adapted from CMAQ v4.7.1 and incorporated the physical processes of advection, diffusion, cloud process and dry deposition. (JPROC: Photolysis Rate Processor; CCTM: CMAQ Chemical Transport Model.)

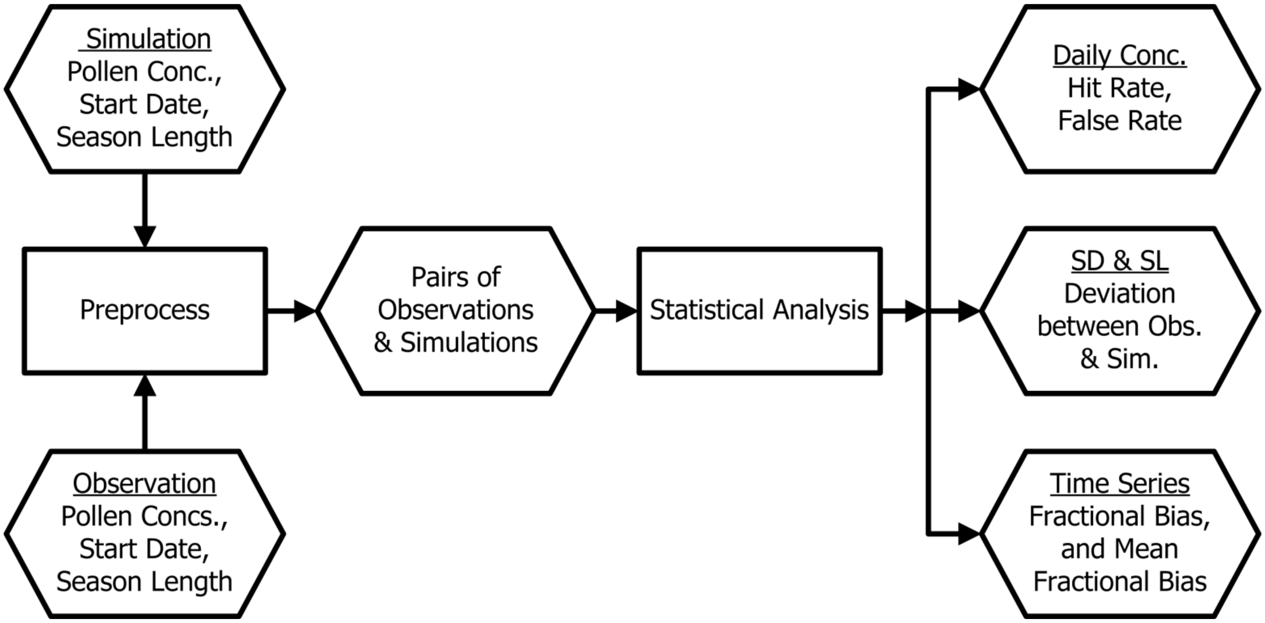

Supplementary Figure 2. Procedures used to evaluate the performance of the CMAQ-Pollen modeling system based on observed pollen count.

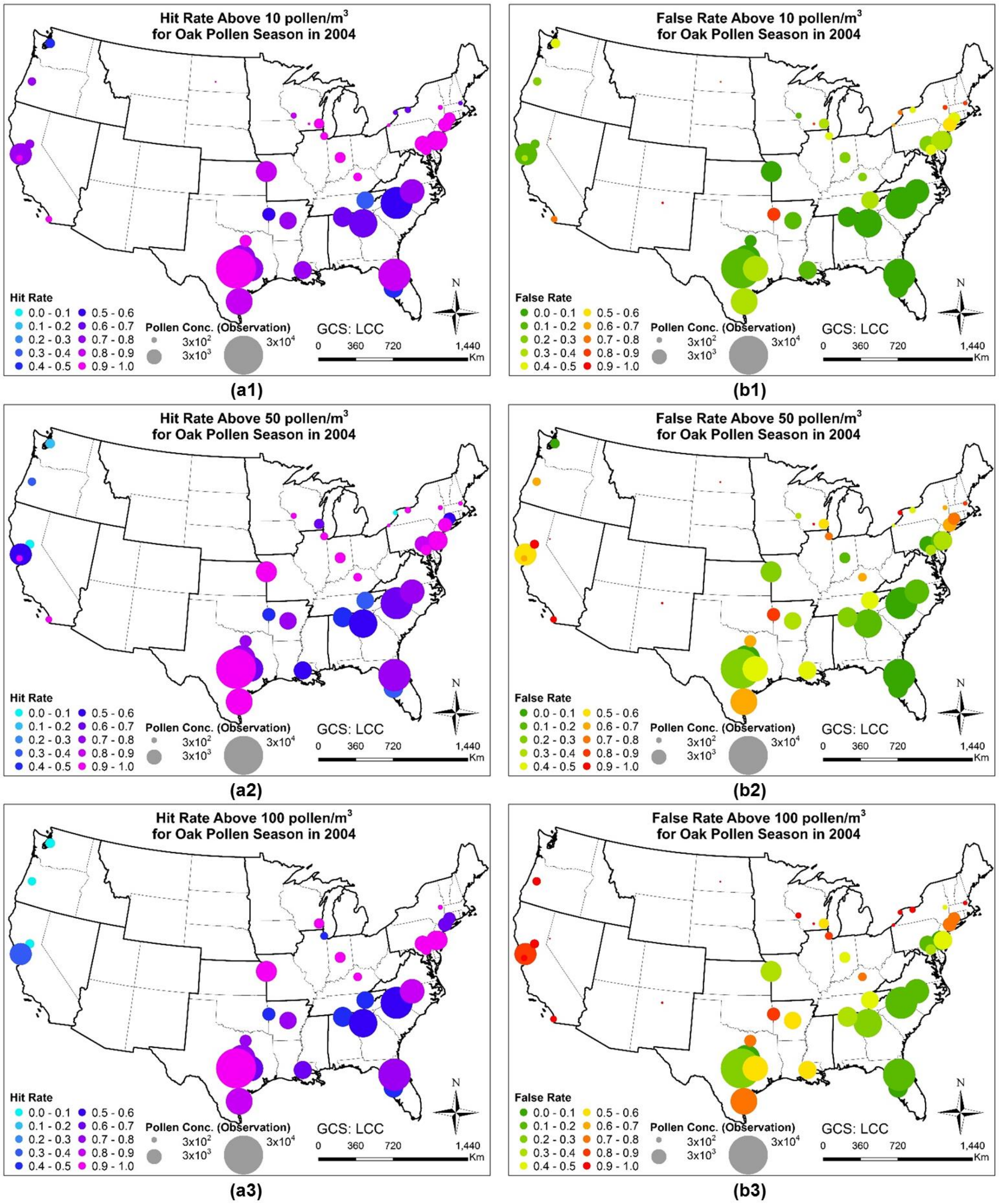

Supplementary Figure 3. Hit and false rates for predicted and observed daily oak pollen concentration during 2004 in the CONUS. The size of the circle indicates the oak pollen abundance at that station. (a1-a3): Hit rates for 10, 50 and 100 pollen grains/m^3, respectively; (b1-b3): False rates for 10, 50 and 100 pollen grains/m^3, respectively.

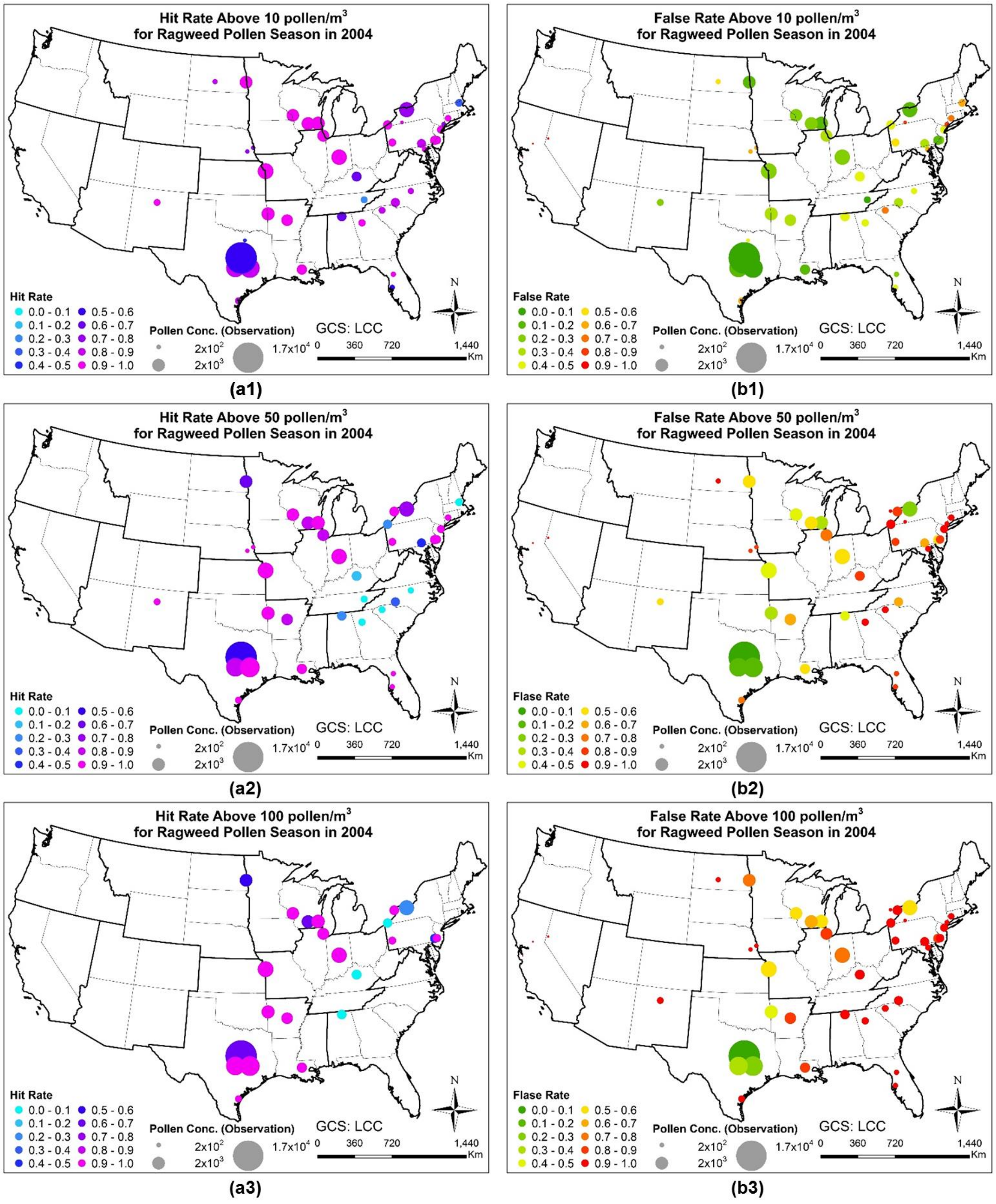

Supplementary Figure 4. Hit and false rates for predicted and observed daily ragweed pollen concentration during 2004 in the CONUS. The size of the circle indicates the ragweed pollen abundance at that station. (a1-a3): Hit rates for 10, 50 and 100 pollen grains/m3, respectively; (b1-b3): False rates for 10, 50 and 100 pollen grains/m^3, respectively.

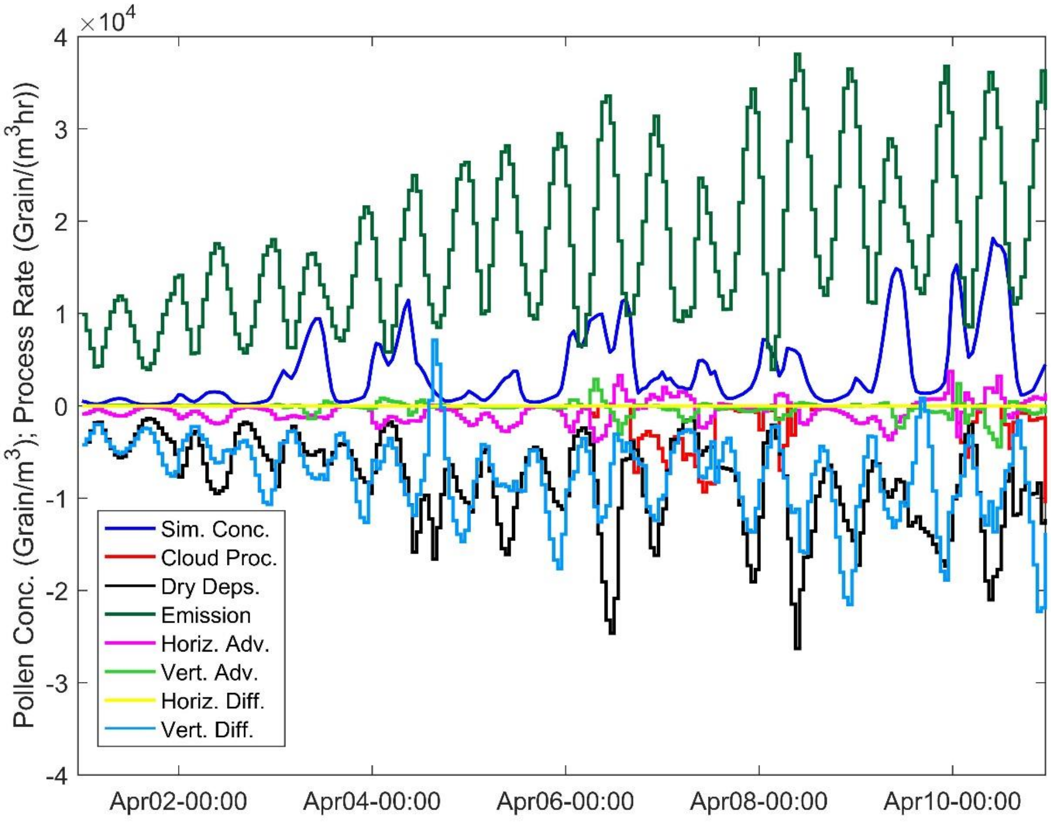

Supplementary Figure 5. Contributions of advection, diffusion, dry deposition and cloud process on modeled hourly oak pollen concentrations for the period between 00:00 UTC April 1 to 23:00 UTC April 10, 2004 in the Atlanta, Georgia. (Sim. Conc.: Simulated Concentration, Cloud Proc.: Cloud Process, Dry Deps.: Dry Deposition, Horiz. Adv.: Horizontal Advection, Vert. Adv.: Vertical Advection, Horiz. Diff.: Horizontal Diffusion, Vert. Diff.: Vertical Diffusion)

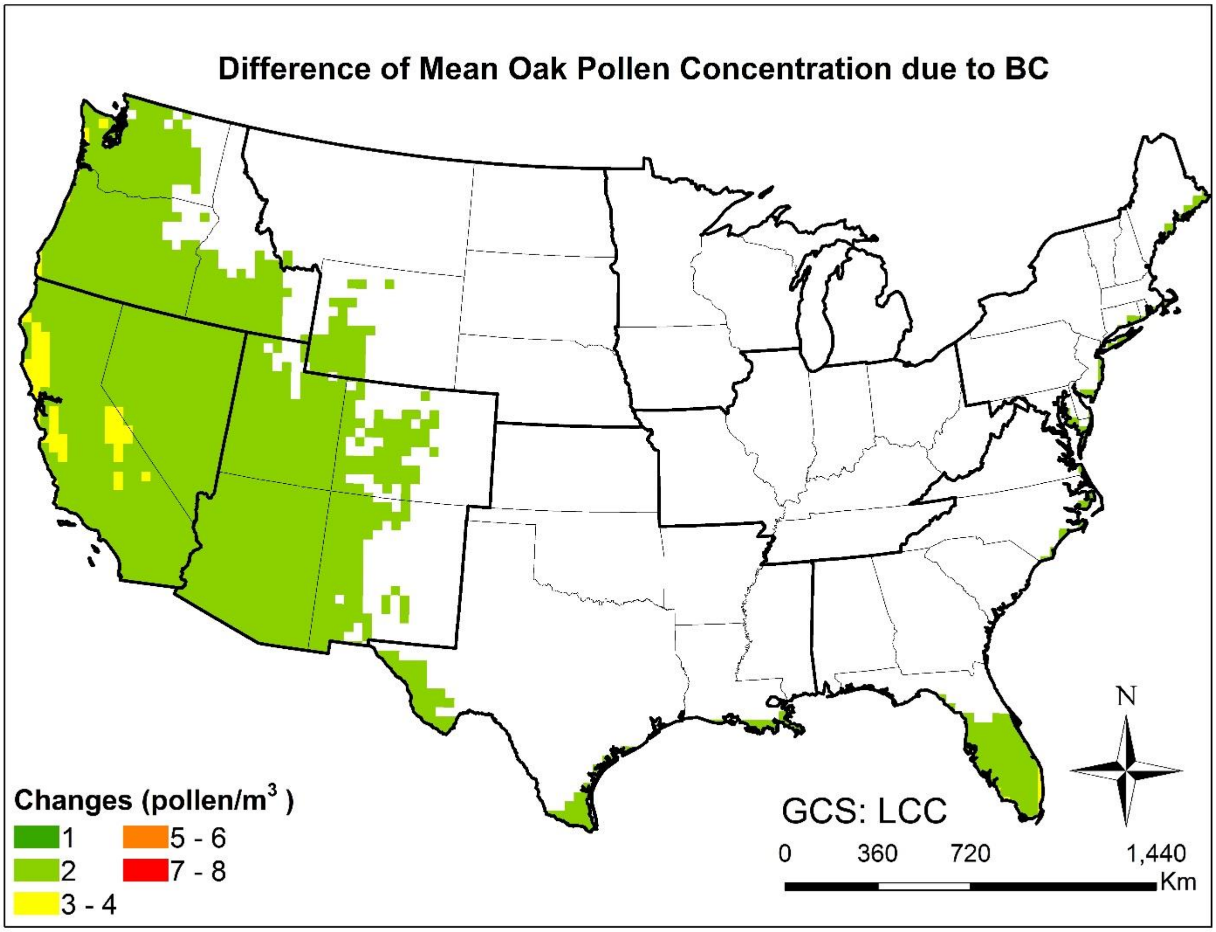

Supplementary Figure 6. Difference in modeled mean oak pollen concentrations in the lowest layer calculated using two different boundary conditions (BC). The default BC was set as 0 pollen grains/m^3, and the other BC was set as 10 pollen grains/m^3.

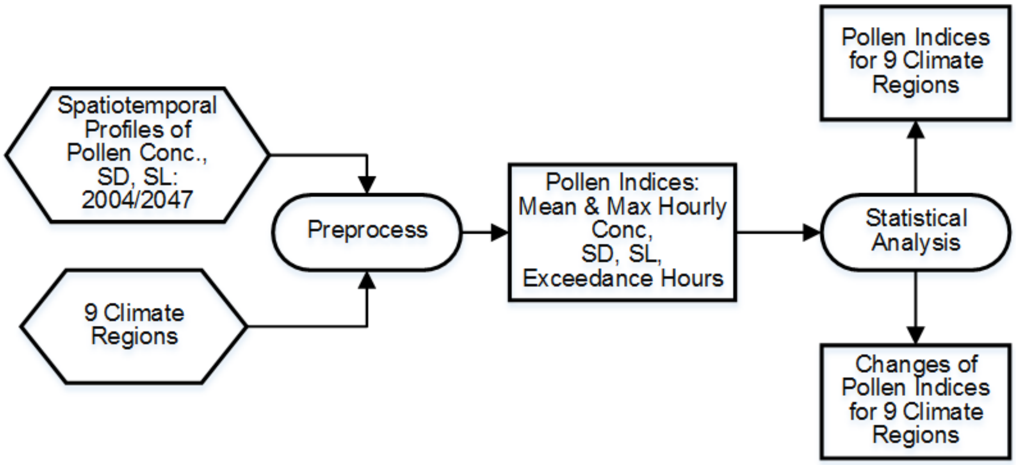

Supplementary Figure 7. Calculation of pollen indices to assess climate change impacts on allergenic pollen.

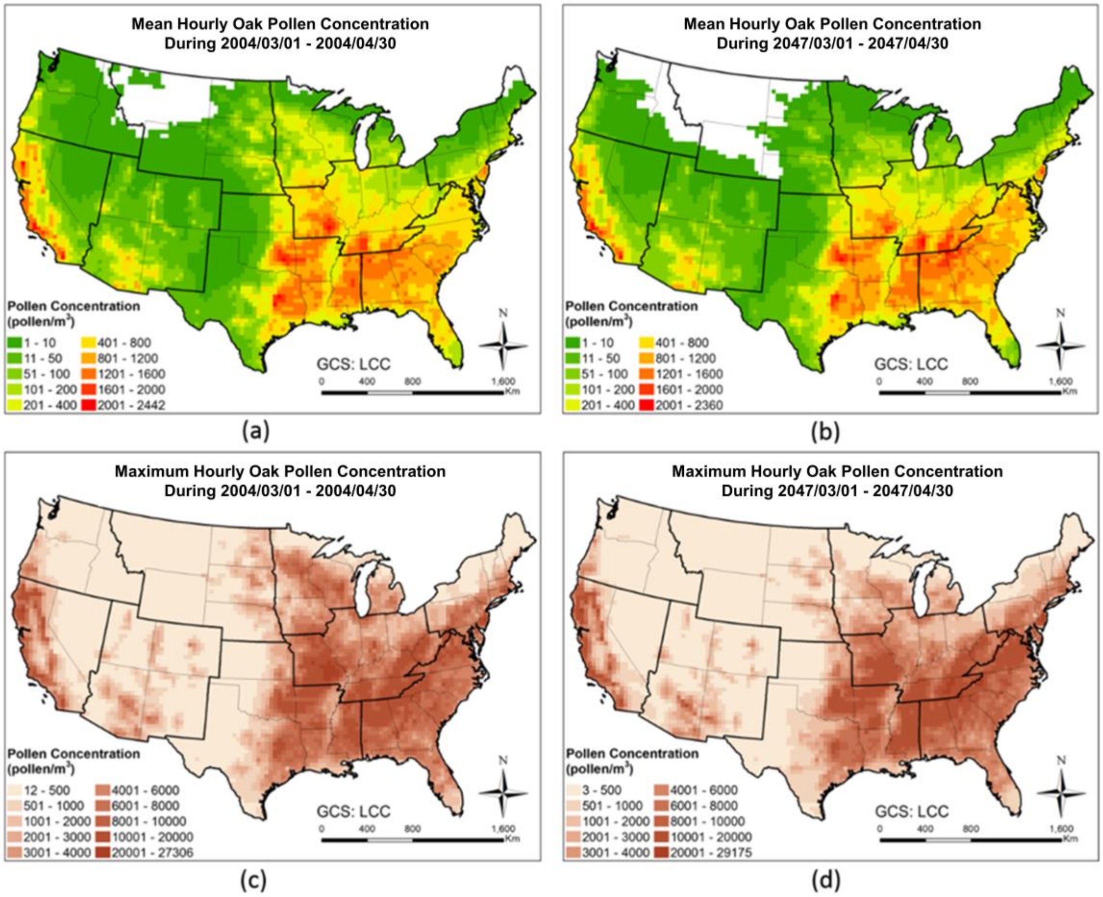

Supplementary Figure 8. Mean (Fig. a and b) and maximum (Fig. c and d) simulated hourly concentrations of oak pollen in 2004 and 2047 using the CMAQ-Pollen modeling system.

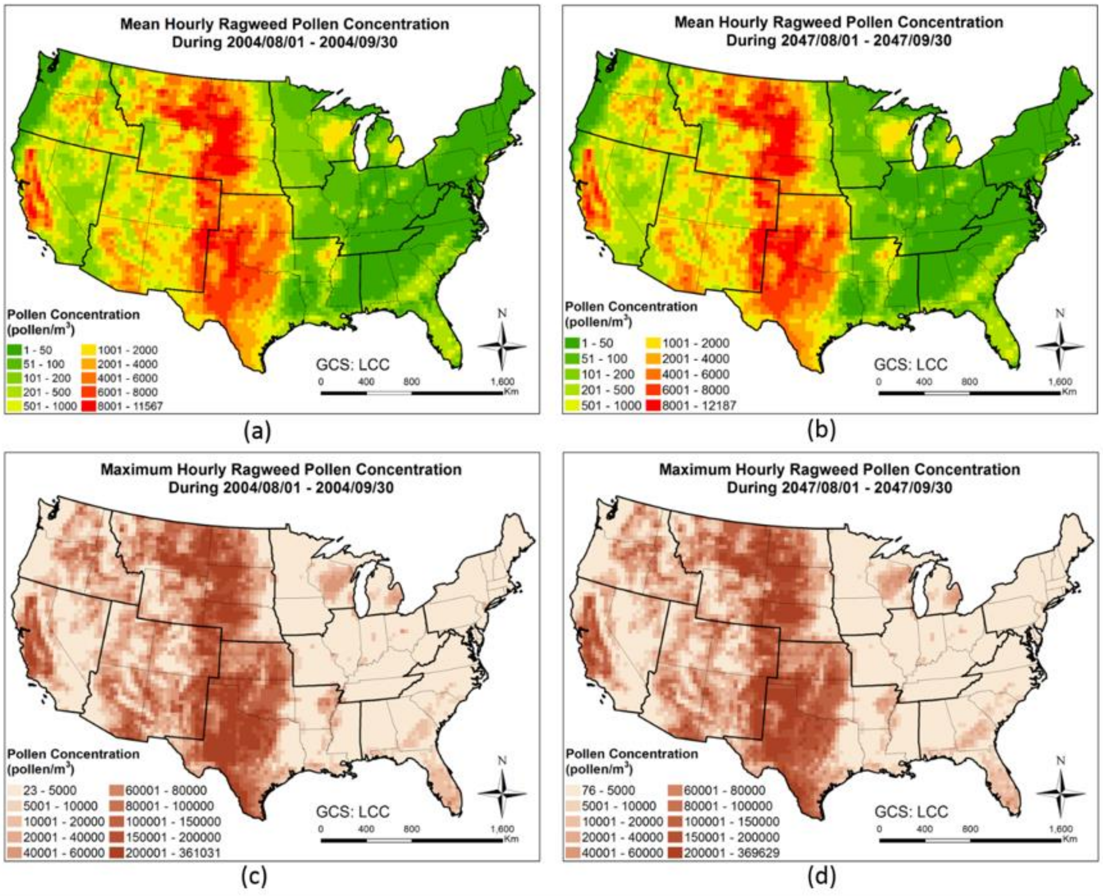

Supplementary Figure 9. Mean (Fig. a and b) and maximum (Fig. c and d) simulated hourly concentrations of ragweed pollen in 2004 and 2047 using the CMAQ-Pollen modeling system.

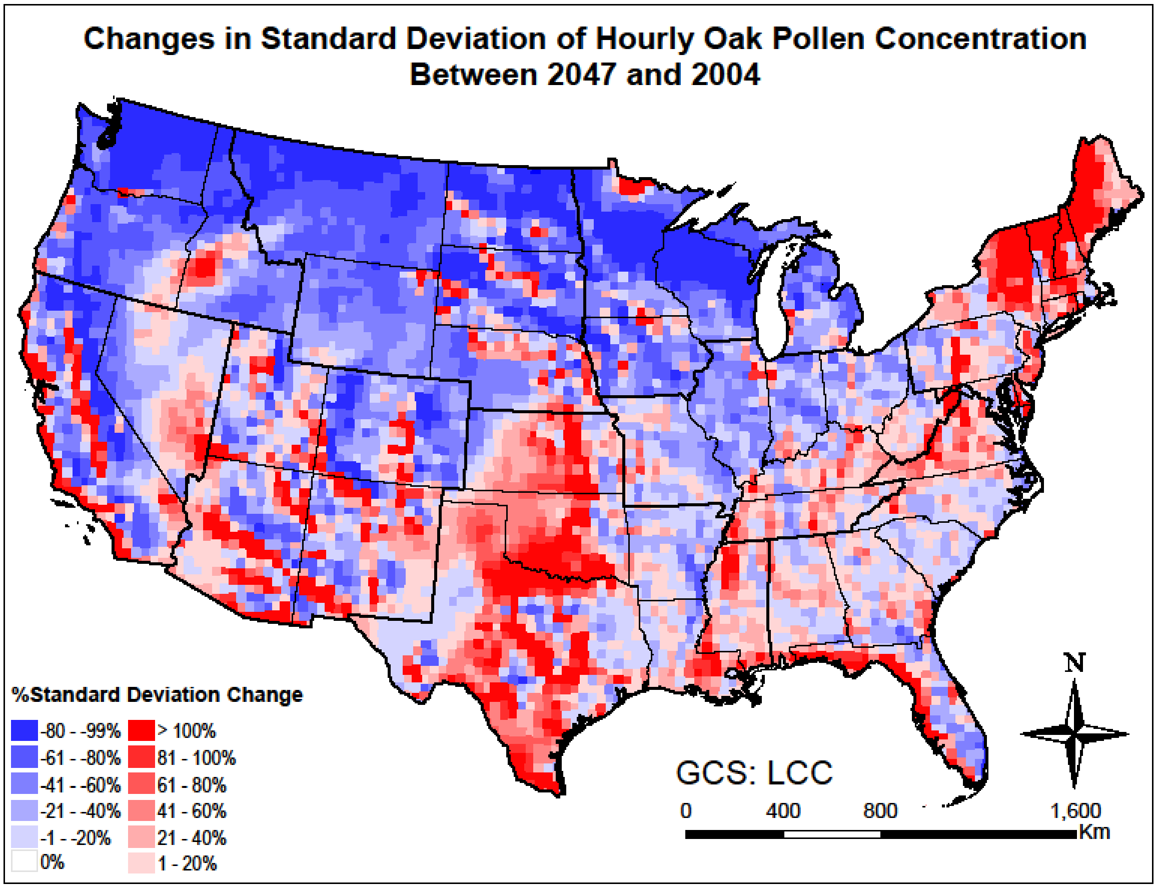

Supplementary Figure 10. Changes in standard deviation of hourly oak pollen concentration between 2047 and 2004 using the CMAQ-Pollen modeling system.

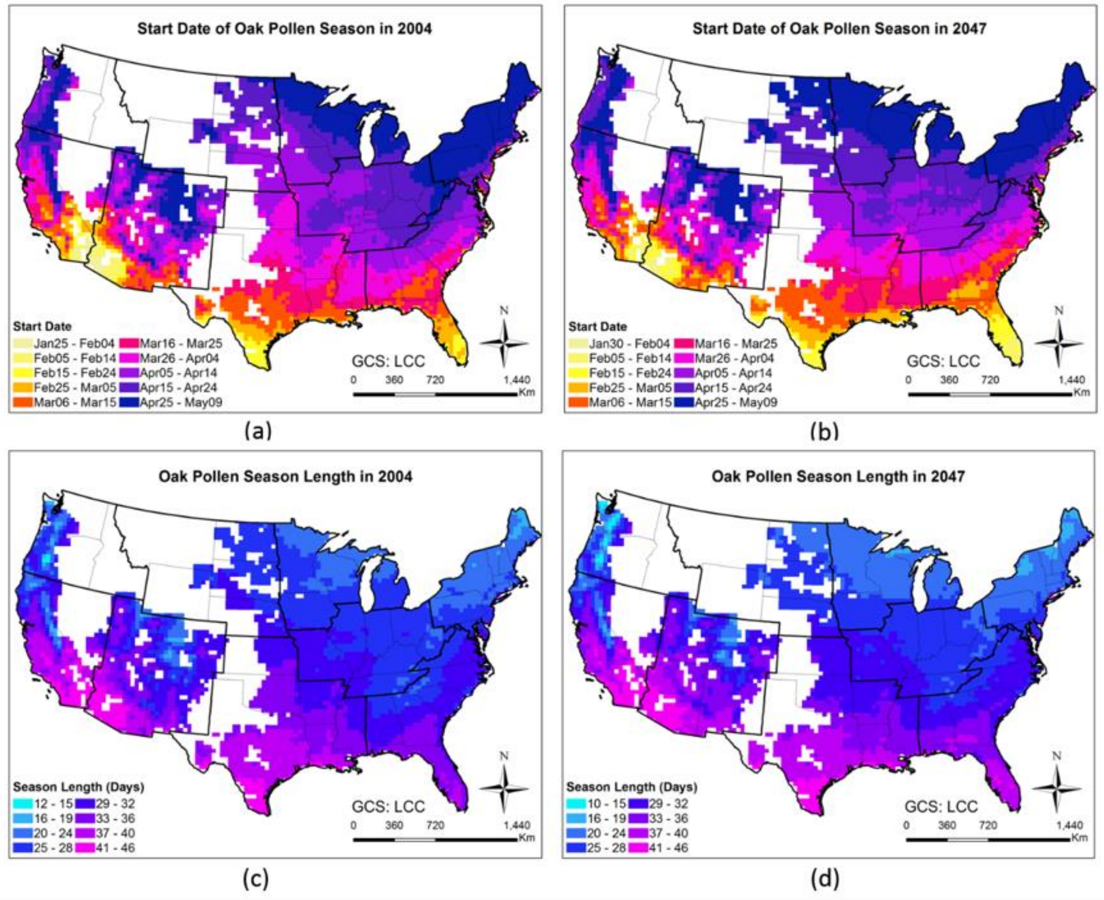

Supplementary Figure 11. Start date (Fig. a and b) and season length (Fig. c and d)) of oak pollen season in 2004 and 2047 simulated using the CMAQ-Pollen modeling system.

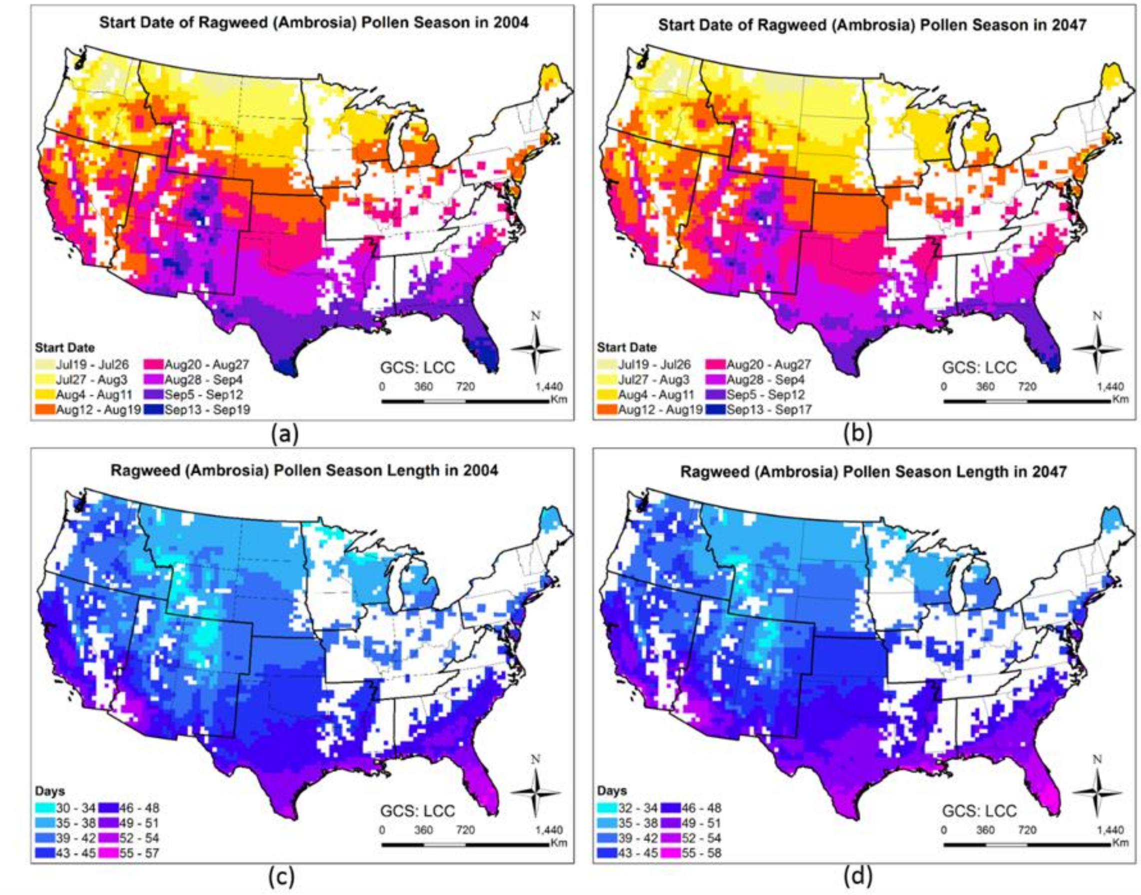

Supplementary Figure 12. Start date (Fig. a and b) and season length (Fig. c and d)) of ragweed pollen season in 2004 and 2047 simulated using the CMAQ-Pollen modeling system.

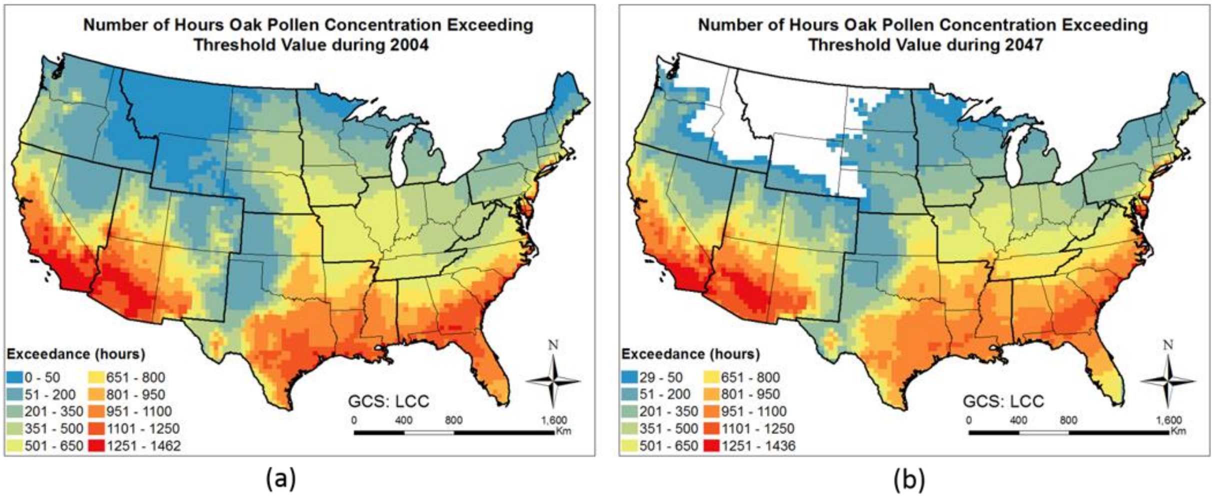

Supplementary Figure 13. Number of hours during which oak pollen concentration exceeds 13 pollen grains/m^3 during 2004 and 2047 simulated using the CMAQ-Pollen modeling system.

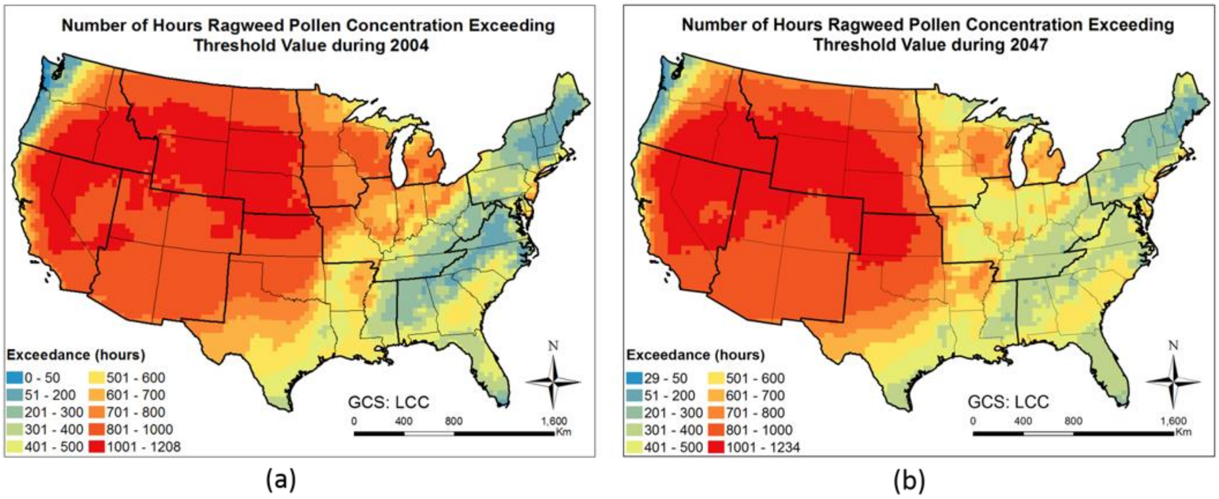

Supplementary Figure 14. Number of hours during which ragweed pollen concentration exceeds 30 pollen grains/m^3 during 2004 and 2047 simulated using the CMAQ-Pollen modeling system.

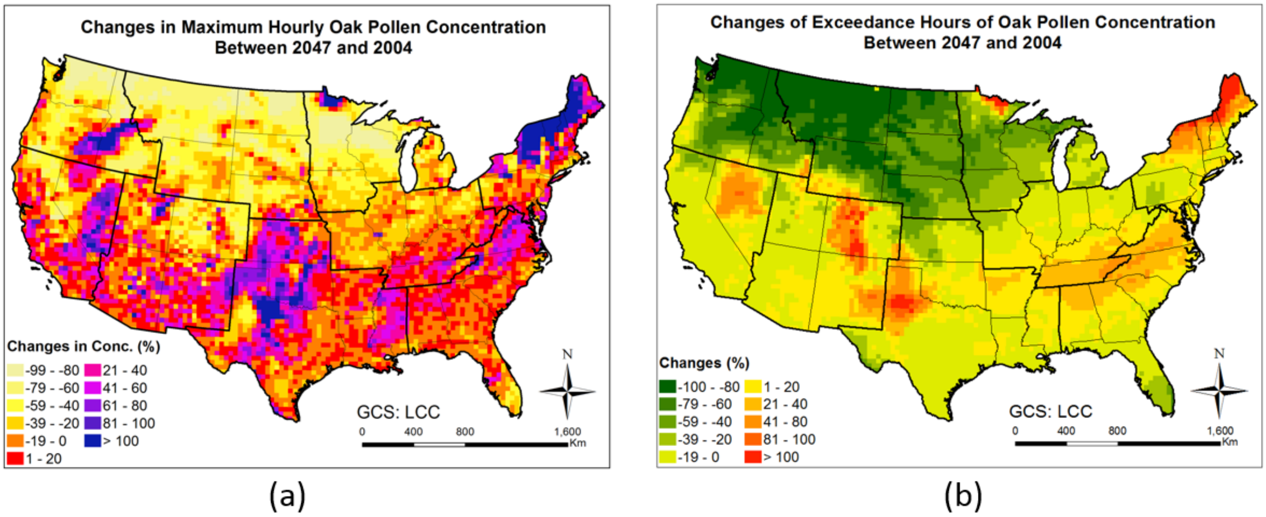

Supplementary Figure 15. Changes in oak pollen season between 2047 and 2004 simulated using the CMAQ-Pollen modeling system. (a) Maximum hourly concentrations, and (b) Exceedance hours.

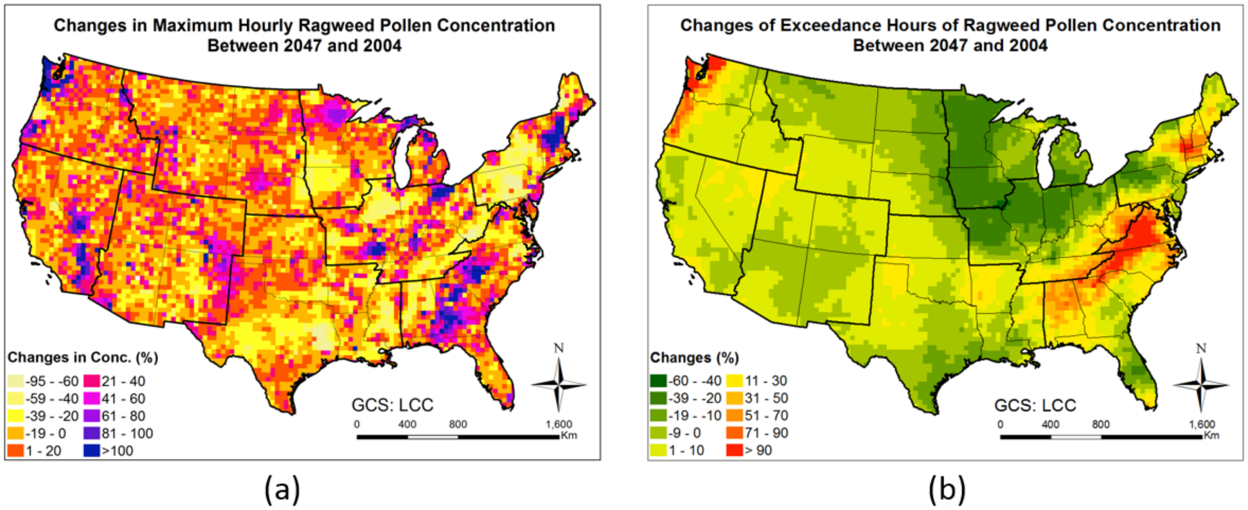

Supplementary Figure 16. Changes in ragweed pollen season between 2047 and 2004 simulated using the CMAQ-Pollen modeling system. (a) Maximum hourly concentrations, and (b) Exceedance hours.

References

1. Prank M, Chapman DS, Bullock JM, Belmonte J, Berger U, Dahl A, et al. An operational model for forecasting ragweed pollen release and dispersion in Europe. Agricultural and Forest Meteorology. 2013;182–183(0):43-53.

2. Zink K, Vogel H, Vogel B, Magyar D, Kottmeier C. Modeling the dispersion of Ambrosia artemisiifolia L. pollen with the model system COSMO-ART. International Journal of Biometeorology. 2012;56(4):669-80.

3. Zhang R, Duhl T, Salam M, House J, Flagan R, Avol E, et al. Development of a regional-scale pollen emission and transport modeling framework for investigating the impact of climate change on allergic airway disease. Biogeosciences. 2014;11(6):1461-78.

4. Nolte CG, Spero TL, Bowden JH, Mallard MS, Dolwick PD. The potential effects of climate change on air quality across the conterminous US at 2030 under three Representative Concentration Pathways. Atmos Chem Phys. 2018;18(20):15471-89.

5. Spero TL, Nolte CG, Bowden JH, Mallard MS, Herwehe JA. The Impact of Incongruous Lake Temperatures on Regional Climate Extremes Downscaled from the CMIP5 Archive Using the WRF Model. J Climate. 2016;29(2):839-53.

6. Morris MD. Factorial sampling plans for preliminary computational experiments. Technometrics. 1991;33(2):161-74.

7. Zhang Y, Bielory L, Cai T, Mi Z, Georgopoulos P. Predicting onset and duration of airborne allergenic pollen season in the United States. Atmospheric Environment. 2015;103(0):297-306.

8. Zhang Y, Bielory L, Mi Z, Cai T, Robock A, Georgopoulos P. Allergenic pollen season variations in the past two decades under changing climate in the United States. Global Change Biology. 2015;21(4):1581-9.

9. Cai T, Zhang Y, Ren X, Bielory L, Mi Z, Nolte CG, et al. Development of a semi-mechanistic allergenic pollen emission model. Science of the Total Environment. 2019;653:947-57.

10. Kawashima S, Takahashi Y. An improved simulation of mesoscale dispersion of airborne cedar pollen using a flowering-time map. Grana. 1999;38(5):316-24.

11. Helbig N, Vogel B, Vogel H, Fiedler F. Numerical modelling of pollen dispersion on the regional scale. Aerobiologia. 2004;20(1):3-19.

12. Pasken R, Pietrowicz JA. Using dispersion and mesoscale meteorological models to forecast pollen concentrations. Atmospheric Environment. 2005;39(40):7689-701.

13. Sofiev M, Siljamo P, Ranta H, Rantio-Lehtimäki A. Towards numerical forecasting of long-range air transport of birch pollen: theoretical considerations and a feasibility study. International Journal of Biometeorology. 2006;50(6):392-402.

14. Schueler S, Schlünzen K. Modeling of oak pollen dispersal on the landscape level with a mesoscale atmospheric model. Environmental Modeling and Assessment. 2006;11(3):179-94.

15. Vogel H, Pauling A, Vogel B. Numerical simulation of birch pollen dispersion with an operational weather forecast system. International Journal of Biometeorology. 2008;52(8):805-14.

16. Efstathiou C, Isukapalli S, Georgopoulos P. A mechanistic modeling system for estimating large scale emissions and transport of pollen and co-allergens. Atmospheric environment (Oxford, England : 1994). 2011;45(13):2260-76.

17. Duhl T, Zhang R, Guenther A, Chung S, Salam M, House J, et al. The Simulator of the Timing and Magnitude of Pollen Season (STaMPS) model: a pollen production model for regional emission and transport modeling. Geoscientific Model Development Discussions. 2013;6(2):2325-68.

18. Siljamo P, Sofiev M, Filatova E, Grewling L, Jager S, Khoreva E, et al. A numerical model of birch pollen emission and dispersion in the atmosphere. Model evaluation and sensitivity analysis. International Journal of Biometeorology. 2013;57(1):125-36.

19. Sofiev M, Siljamo P, Ranta H, Linkosalo T, Jaeger S, Rasmussen A, et al. A numerical model of birch pollen emission and dispersion in the atmosphere. Description of the emission module. International Journal of Biometeorology. 2013;57(1):45-58.

20. Sofiev M, Berger U, Prank M, Vira J, Arteta J, Belmonte J, et al. MACC regional multi-model ensemble simulations of birch pollen dispersion in Europe. Atmos Chem Phys Discuss. 2015;15(6):8243-81.

21. Jeon W, Choi Y, Roy A, Pan S, Price D, Hwang M-K, et al. Investigation of primary factors affecting the variation of modeled oak pollen concentrations: a case study for Southeast Texas in 2010. Asia-Pacific Journal of Atmospheric Sciences. 2018;54(1):33-41.
